# *Arabidopsis* root responses to salinity depend on pectin modification and cell wall sensing

**DOI:** 10.1242/dev.200363

**Published:** 2022-06-17

**Authors:** Nora Gigli-Bisceglia, Eva van Zelm, Wenying Huo, Jasper Lamers, Christa Testerink

**Affiliations:** Laboratory of Plant Physiology, Wageningen University and Research, Wageningen 6708 PB, The Netherlands

**Keywords:** Salt stress responses, Plant cell wall signaling, Pectin modifications, Cell wall integrity, *Catharanthus roseus*, Receptor-like kinase 1 like

## Abstract

Owing to its detrimental effect on plant growth, salinity is an increasing worldwide problem for agriculture. To understand the molecular mechanisms activated in response to salt in *Arabidopsis thaliana*, we investigated the *Catharanthus roseus* receptor-like kinase 1-like family, which contains sensors that were previously shown to be involved in sensing the structural integrity of the cell walls. We found that *herk1 the1-4* double mutants, lacking the function of HERKULES1 (HERK1) and combined with a gain-of-function allele of THESEUS1 (THE1), strongly respond to salt application, resulting in an intense activation of stress responses, similarly to plants lacking FERONIA (FER) function. We report that salt triggers pectin methyl esterase (PME) activation and show its requirement for the activation of several salt-dependent responses. Because chemical inhibition of PMEs alleviates these salt-induced responses, we hypothesize a model in which salt directly leads to cell wall modifications through the activation of PMEs. Responses to salt partly require the functionality of FER alone or HERK1/THE1 to attenuate salt effects, highlighting the complexity of the salt-sensing mechanisms that rely on cell wall integrity.

## Abstract (precis)

**Summary:** Salt-triggered activation of pectin methyl esterase changes pectin in *Arabidopsis*, inducing at least two pathways: a CrRLK1L-dependent pathway downregulating salt stress responses and a CrRLK1L-independent pathway that activates downstream signaling.

## INTRODUCTION

Salinity is detrimental to plant growth as salt simultaneously causes both ionic and osmotic stress, triggering subsequential general and specific signaling responses (Lamers et al., 2020; van Zelm et al., 2020). Recently, the role of plant cell wall (CW) modifications as possible factors in regulating salt stress responses have gained special interest (Feng et al., 2018). In plants, CWs act as a barrier that protects plants and functions as structural support for the whole plant body (Underwood, 2012). In the primary CWs of young *Arabidopsis* plants, the main load-bearing component is cellulose, made of β-1,4-linked glucose monomers. Cellulose fibrils are connected with pectin, a polysaccharide consisting mainly of a backbone of homogalacturonan (HG, α-1,4-linked galacturonic acid units), which can be found in methyl-esterified or de-methyl-esterified forms (Peaucelle et al., 2011; Pelloux et al., 2007). Together with cellulose and hemicellulose, pectin is described to be essential for regulating cell expansion and elasticity (reviewed by Gigli-Bisceglia et al., 2020).

CWs are subjected to controlled modifications that allow the cell to extend (reviewed by Vaahtera et al., 2019). CW modifications also occur in response to both biotic and abiotic stresses and these structural changes are perceived by the plants. CW perception seems to be mediated by different cell surface receptors [i.e. wall-associated kinases (WAKs), leucine-rich repeat receptor-like kinases (LRR-RLKs), LysM receptor-like kinases (LyKs), leucine-rich repeat extensins (LRXs) and the *Catharanthus roseus* receptor-like kinase 1-like (CrRLK1L) family (reviewed by Bacete et al., 2018)]; however, it is unclear what the sensors can perceive. In response to biotic stresses, CW fragments can be released and sensed (through WAK or LyK family members) (De Lorenzo et al., 2019; Kohorn, 2016; Mélida et al., 2018) after active degradation of the CWs, and a similar mechanism that is activated in response to abiotic stress has not yet been described (Gigli-Bisceglia and Testerink, 2021). It has been suggested that CW perception might be dependent on the perception of the mechanical distortion between the CW and the plasma membrane (PM), and/or modification of the CW composition leading to a change in CW strength. Receptors responsible for triggering intracellular downstream signals in response to turgor pressure-driven CW-dependent mechanical distortions have been identified, and a process called CW integrity (CWI) maintenance, originally identified in yeast, was found and characterized in plants (Dupres et al., 2009; Levin, 2011; Rui and Dinneny, 2020). Even if a relatively large number of cell surface receptors have been proposed to function as CW sensors, evidence on their role is limited to a few families. Members of the CrRLK1L family have been described to play a role in a plethora of mechanisms related to CW remodeling (e.g. cellulose impairment, pathogen infections and fertility) (Nissen et al., 2016). CrRLK1Ls contain two malectin domains, proposed to bind complex carbohydrates and/or small extracellular peptides called rapid alkalinization factors (RALFs) (Abarca et al., 2021; Schallus et al., 2008).

In *Arabidopsis*, 17 CrRLK1Ls have been identified and, in some cases, their functional roles have been extensively studied. For example, FERONIA (FER) appears to be vital in multiple aspects of plant life (e.g. fertility, mechanical stress and immune responses), whereas THESEUS1 (THE1), HERKULES 1/2 (HERK1/2) and CURVY (CVY1) appear to be essential for cell elongation and cell morphology-related processes (Guo et al., 2009; Nissen et al., 2016; Voxeur and Höfte, 2016). THE1 has been described to be responsible for sensing CW alterations and mechanical stress responses upon cellulose impairment (Engelsdorf et al., 2018; Gigli-Bisceglia et al., 2018b), but at the same time appears to be essential for controlling the mechanical properties of cell walls that affect turgor pressure in plants (Bacete et al., 2022). Several proteins have been characterized to play a role in CWI signaling, including stretch-activated PM-localized Ca^2+^ channels (MCAs) and the NITRATE REDUCTASE 1 and 2 (NIA1/2), the latter having been recently described to play an essential role in CW damage (CWD)-dependent cell cycle regulation (Engelsdorf et al., 2018; Gigli-Bisceglia et al., 2018b; 2020; Vaahtera et al., 2019).

Interestingly, many of the CWI players have been found to overlap with regulators of the salt stress response. This is the case for the LRR-RLKs MALE DISCOVERER 1-INTERACTING RECEPTOR-LIKE KINASE 2 (MIK2/KISS), FEI1/2 and FERONIA, and also for NIAs and MCAs (Feng et al., 2018; Harpaz-Saad et al., 2012; Shabala et al., 2015; Van der Does et al., 2017; Xie et al., 2013). Intriguingly, although mutants for *mik2*, *fei1/2*, *nia1/2* and *mca1/2* were reported to be highly sensitive to salt application, their responses to chemically induced cellulose impairment varied, with *fei1/2* being extremely sensitive and *mik2*, *nia1/2* and *mca1* being partly or almost completely insensitive to cellulose inhibition (Engelsdorf et al., 2018; Gigli-Bisceglia et al., 2018b; Harpaz-Saad et al., 2012; Shabala et al., 2015; Van der Does et al., 2017; Xie et al., 2013). However, it remains unknown how and why CWI and salt-induced signaling overlap.

Extensive studies performed on the CrRLK1L FER seem to link CW architecture and responses to salt stress, highlighting the importance of the CrRLK1L-mediated CW integrity maintenance during abiotic stress (Feng et al., 2018; Zhao et al., 2021, 2018). In fact, *fer-4* mutants display cell bursting and severe root-cell swelling in the presence of high salt concentrations and this phenotype was associated with a *fer*-dependent enhanced CW softening (Feng et al., 2018). Treatment with Ca^2+^ and boron/borate ions, proposed to enhance pectin-pectin crosslinks, can complement FER-dependent salt-induced phenotypes, suggesting that salt application might alter pectin architecture (Feng et al., 2018). Intriguingly, FER's malectin domain can bind pectin *in vitro*, likely having physical preferences for de-methyl-esterified HG (Feng et al., 2018; Lin et al., 2022). The FER-pectin interaction seems to signal the CW status intracellularly through the functionality of the ROP-GTPases (Lin et al., 2022). However, although the FER-pectin interaction seems essential to determine cell shape and morphogenesis (Lin et al., 2022), whether FER interacts with pectin during salt stress is unknown. Other protein kinases, such as the WAKs or the PROLINE-RICH EXTENSIN-LIKE RECEPTOR KINASE 4 (PERK4), have been shown to directly bind pectin (Bai et al., 2009; Kohorn and Kohorn, 2012), but a role in salt stress has not been described. Whether the combination of small peptide perception and CW sensing is essential for the process is still unknown. It was recently shown that the ELICITOR PLANT PEPTIDE 3 (PEP3) alleviates salt-triggered phenotypes (Nakaminami et al., 2018) and, in response to CW damage, accumulates and inhibits downstream responses (Engelsdorf et al., 2018).

To address the role of CW modifications in salt stress responses, we first investigated early and late signaling, gene expression and physiological events as well as CW modification in *Arabidopsis* seedlings upon salt stress. We report that salt increases pectin methyl esterase (PME) activity by enhancing the degree of de-methyl-esterified HG. This event appears to be partly responsible for the activation of early stress responses [i.e. MITOGEN PROTEIN KINASE 6 (MPK6) phosphorylation] and salt-dependent marker gene induction, because the application of chemicals that inhibit PME activity *in vitro* alleviates many of the salt-dependent responses *in vivo*. Moreover, although loss-of-function mutants (lacking THE1 or HERK1 alone) show wild-type-like salt stress responses, plants lacking HERK1 in combination with a truncated gain-of-function allele version of THE1 (*herk1 the1-4* mutants; Guo et al., 2009) show severe salt sensitivity. Similarly to FER, HERK1/THE1 seem to be partially required for dampening the salt-induced activation of MPK6, as higher activation of MPK6 is observed in *fer*-4 and *herk1 the1-4* mutants, which is consistent with the higher expression of salt-induced, CWD-dependent marker genes in these mutants. Collectively, we hypothesize a scenario in which salt-dependent PME activation changes the structural integrity and the mechanical properties of the CW, and that this event would be sufficient to activate at least two branches of the salt-activated signaling; one independent of and one dependent on the function of the CrRLK1Ls that are responsible for negatively regulating different downstream responses to salt.

## RESULTS

### *herk1 the1-4* and *fer-4* mutants are affected in early responses to salt

**Fig. 1.**
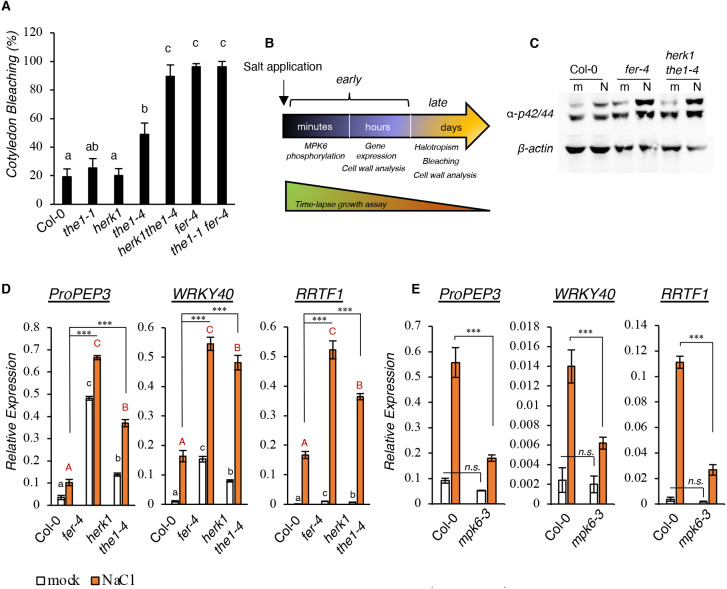
***herk1 the1-4* and *fer-4* mutant lines show enhanced early responses to salt stress.** (A) Cotyledon bleaching analysis in 10-day-old seedlings germinated in 150 mM NaCl plates. Values, expressed as percentages, are the average of four independent experiments. Error bars represent s.e.m. One-way ANOVA and Tukey's HSD (α=0.05) were used to compare differences between genotypes within one treatment condition (salt). (B) Timeline of the treatments performed in this study. (C) Seven-day-old seedlings of the Col-0, *fer-4* and *herk1 the1-4* lines were treated for 15 min with water (mock, ‘m’) or 100 mM NaCl (‘N’). Phosphorylation of MPKs was detected by immunoblotting. Equal loading was checked using an anti-β-actin antibody. Similar results were obtained in three biological replicates. (D) Seedlings as in C were treated for 1 h and the expression of *PROPEP3*, *WRKY40* and *RRTF1* was determined by qRT-PCR and represented as the average of three biological replicates (*n*=3). Error bars indicate s.d. Statistical comparisons were made using one-way ANOVA and Tukey's HSD (α=0.05) considering the effect of the same treatment in different genotypes (black lowercase letters for mock, red uppercase letters for salt treatment). Comparisons between salt-treated wild-type seedlings and other genotypes were made by using multiple two-tailed unpaired *t*-tests coupled with Benjamini–Hochberg false discovery rate correction. ***P<0.001. (E) Seven-day-old seedlings of the Col-0 and *mpk6-3* lines were treated as in D. Gene expression analysis was performed after 1 h water (mock) or 100 mM NaCl treatment. *n*=3. Error bars indicate s.d. Asterisks show statistical comparisons between NaCl-treated Col-0 or *mpk6-3* according to multiple unpaired two-tailed *t*-tests and Benjamini–Hochberg correction. ***P<0.001; n.s., not significant.

To investigate whether other CrRLK1Ls, apart from *fer-4*, are disturbed by salt application (Feng et al., 2018; Zhao et al., 2018), a collection of available mutants from the CrRLK1L protein family previously implicated in CWI sensing (Table S1) were directly germinated and grown in the presence or absence of 150 mM NaCl for 10 days (Fig. S1A). No bleaching was observed in control plates and the differences between the genotypes were only quantified in salt-containing plates (Fig. 1A). After 10 days of growth, *fer-4* mutants showed higher rates of cotyledon bleaching, whereas both *herk1* and *the1-1* single knockout (KO) mutants displayed salt-induced cotyledon bleaching that was comparable with that observed in salt-grown wild-type (wt) controls. However, slightly more bleaching was discovered in the single *the1-4* mutant, which expresses the truncated THE1 protein and was previously described as a gain-of-function allele of THE1 (Merz et al., 2017). Interestingly, the double *herk1 the1-4* mutant showed enhanced bleaching comparable with that in *fer-4* (Fig. 1A), suggesting that a combination of HERK1 and THE1 is required to regulate salt-dependent sensitivity. Genetically combining the *the1-1* and *fer-4* mutants led to the same bleaching phenotype as *fer-4* single mutants (Fig. 1A; Fig. S1A), suggesting that the FER-dependent salt-induced phenotype cannot be alleviated by impairing the functionality of the CWD sensor THE1. To investigate further the role of CrRLK1L mutants in salt-induced signaling, different experimental setups have been employed to analyze early (MPK6 phosphorylation and gene expression) and late (root halotropism, cotyledon bleaching and CW composition) responses to salt stress (Fig. 1B).

To understand whether the salt-triggered hypersensitivity to salt observed in *fer-4* as well as in *herk1 the1-4* are the result of enhanced salt-dependent signaling, we analyzed early responses upon salt application. In salt-treated plants, one of the early responses is the phosphorylation of MPK6 (Yu et al., 2010). In plants and animals, mitogen-activated protein kinase (MAPK) cascades are initiated by a specific stimulus that triggers the activation of MAPK kinase kinases (MAPKKKs), phosphorylating a MAPK kinase (MAPKK), and in turn leading to the activation of MAPKs, also known in *Arabidopsis* as mitogen protein kinases (MPKs) (Morrison, 2012). By using a well-characterized and widely used antibody (Bigeard and Hirt, 2018; Galletti et al., 2011; Savatin et al., 2014), which recognizes the phosphorylated form of both *Arabidopsis* MPK6 and MPK3, we analyzed salt-induced MPK6 phosphorylation upon 15 min of salt treatment. We first performed control experiments in a previously characterized MPK6 allele (*mpk6-3*) (Yu et al., 2010) to confirm the specificity of the antibody (Fig. S1B). Then, we used the same setup to perform the analysis in the mutants. Both *fer-4* and *herk1 the1-4* mutant lines showed a stronger MPK6 activation compared with NaCl-treated wt seedlings (Fig. 1C; Fig. S1C). Interestingly, similar to the bleaching phenotype, although *herk1* and *the1*-*1* did not show a difference with salt-treated wt control, *the1-4* displayed enhanced salt-induced MPK6 activation compared with the wt, which was less evident compared with the corresponding double *herk1 the1-4* mutants or *fer-4* (Fig. S1C).

To investigate whether the lack of FER or lack of HERK1 coupled with the THE1 gain-of-function allele had an effect on salt-induced gene expression levels, we selected three salt-induced marker genes (Engelsdorf et al., 2018; Nakaminami et al., 2018; Saijo et al., 2021; Soliman and Meyer, 2019) and checked their expression in response to salt. We chose *PROPEP3*, *WRKY40* and *RRTF1* because they are strongly induced within 1 h from salt application and because they have been described to be responsive to a wide number of salt-dependent modifications, such as CW changes, abscisic acid (ABA)-dependent signaling and reactive oxygen species (ROS) (Engelsdorf et al., 2018; Nakaminami et al., 2018; Saijo et al., 2021; Soliman and Meyer, 2019) (Table S2). We treated wt, *fer-4* and *herk1 the1-4* seedlings for 1 h with salt and found that the expression levels of salt-responsive genes were higher in response to salt in the mutant lines compared with the wt controls (Fig. 1D). Similar to the cotyledon bleaching rates, *the1-4*, but not *the1-1*, showed enhanced transcriptional regulation of salt marker genes compared with salt-treated wt seedlings (Fig. S1D). To explain the enhanced salt stress responses of *herk1 the4*, we analyzed *FER* transcripts, but no differences were detected when comparing the expression levels with the wt controls (Fig. S1E), likely suggesting that the observed *herk1 the4* phenotypes are not caused by altered FER expression.

As *mpk6-3* loss-of-function mutants were reported to show reduced root growth inhibition in response to salt stress (Han et al., 2014), likely associated with reduced salt sensitivity, we analyzed the expression pattern of the selected gene markers in the *mpk6-3* mutant and observed that MPK6 is, in part, responsible for their full induction (Fig. 1E). The results suggest that the enhanced MPK6 activation observed in *fer-4* and *herk1 the1-4* might also be responsible for their amplified susceptibility to salt. Recently, it was reported that ectopic overexpression of *RRTF1* is detrimental to plant salt tolerance (Soliman and Meyer, 2019). This is consistent with our observations in which the higher *RRTF1* expression pattern seen in both salt-treated *fer-4* or *herk1 the1-4* mutants is linked with higher plant salt susceptibility (Fig. 1D; Fig. S1D).

### *herk1 the1-4* show *fer-4*-like root growth inhibition dynamics after salt application

**Fig. 2.**
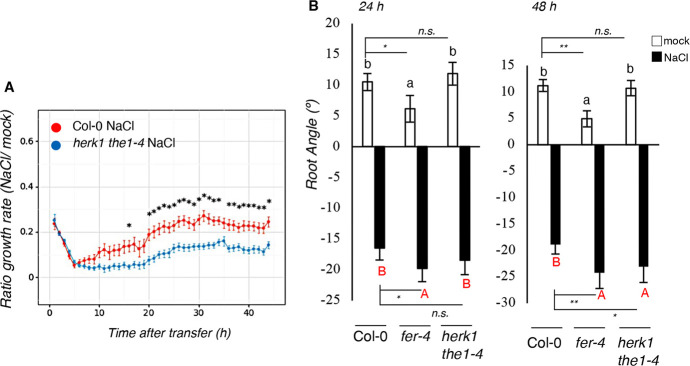
***herk1 the1-4* and *fer-4* mutant lines show enhanced late responses to salt stress.** (A) Root growth rate of 7-day-old Col-0 and *herk1 the1-4* seedlings. Each of the dots represent the average of three independent experiments (*n*=45 per treatment, per genotype). Statistical comparisons were performed using a Levene's test, followed by two-tailed, unpaired *t*-test; *P<0.05. (B) Four-day-old seedlings of the Col-0, *fer-4* and *herk1 the1-4* lines were subjected to an NaCl gradient and root angle was analyzed. Histograms represent the average root angle of three independent experiments, error bars represent s.e.m. One-way ANOVA and Tukey's HSD (α=0.05) were used to compare differences between one treatment in the three genotypes (black lowercase letters for mock, red uppercase letters for NaCl treatment). *n*=150. Multiple two-tailed, unpaired *t*-test comparisons according to Benjamini–Hochberg correction analyzed between mock treatments and salt treatments at 24 and 48 h are shown. *P<0.05; **P<0.01; n.s., not significant.

Next, because *fer-4* was previously reported to show defective root growth upon salt application (Feng et al., 2018), we grew double mutant *herk1 the1-4* seedlings in vertical plates and transferred them after 7 days to plates containing homogeneous NaCl or no NaCl (control). To obtain a complete overview of both salt-triggered early and late effects, salt-induced root growth inhibition was traced over 42 h in a specialized growth chamber and time-lapse photography-based analyses were performed (Fig. 2A). Previously, it has been reported that wt seedlings subjected to salt stress display a multi-phasic trend of growth, which is slowly inhibited within hours from initial salt application (quiescent phase) followed by a recovery phase (Geng et al., 2013). Compared with the wt, *herk1 the1-4* mutant seedlings showed, similar as reported for *fer-4* (Feng et al., 2018), reduced root elongation rate upon salt application, which was associated with an impaired recovery upon induction of the quiescent phase (Fig. 2A).

To evaluate whether HERK1/THE1 as well as FER also regulate other salt responses, we analyzed later responses, such as halotropism and salt tolerance. When facing a salt gradient, plant roots bend to avoid high salt concentrations, a phenotype referred to as halotropism, a mechanism that was shown to be dependent on auxin distribution (Deolu-Ajayi et al., 2019; Galvan-Ampudia et al., 2013; van den Berg et al., 2016). Wild-type, *fer-4* and *herk1 the1-4* double-mutant seedlings were subjected to diagonal (mock or salt) gradients and the root-bending angle was measured 24 h and 48 h after salt application. Compared with the wt, *fer-4* exhibited slightly enhanced halotropism-triggered root bending after 24 h, whereas *herk1 the1-4* only displayed differences after 48 h (Fig. 2B; individual biological replicates/root elongation rates are shown in Fig. S2A,B). The salt-dependent root halotropism responses detected in *mpk6-3* (and shown as result of two independent experiments in Fig. S2C) displayed no significant differences compared with the wt controls, suggesting that the mild halotropism defects observed in the *fer-4* and *herk1 the1-4* mutants were MPK6 independent. To evaluate whether the higher salt sensitivity observed in the double *herk1 the1-4* mutant was also present in adult plants, we investigated the responses to salt in soil-grown plants (Fig. S2D). For this assay, Col-0, *fer-4* and *herk1 the1-4* plants were watered once with Milli-Q water or treated with NaCl (75 mM), and then the fresh weight of rosette was measured at the end of the treatment. Fresh weight quantification showed a significant difference between salt-treated wt and the two mutant lines (Fig. S2E), whereas reduced biomass was also observed in the control conditions (Fig. S2F). Taken together, our results show that the *herk1 the1-4* mutant exhibits a similarly enhanced salt sensitivity as that detected in *fer* mutants, not only for early stress responses (Fig. 1), but also in growth dynamics and root elongation upon salt stress application (Fig. 2).

### Salt-induced homogalacturonan de-methyl esterification is required for full activation of salt-triggered responses

**Fig. 3.**
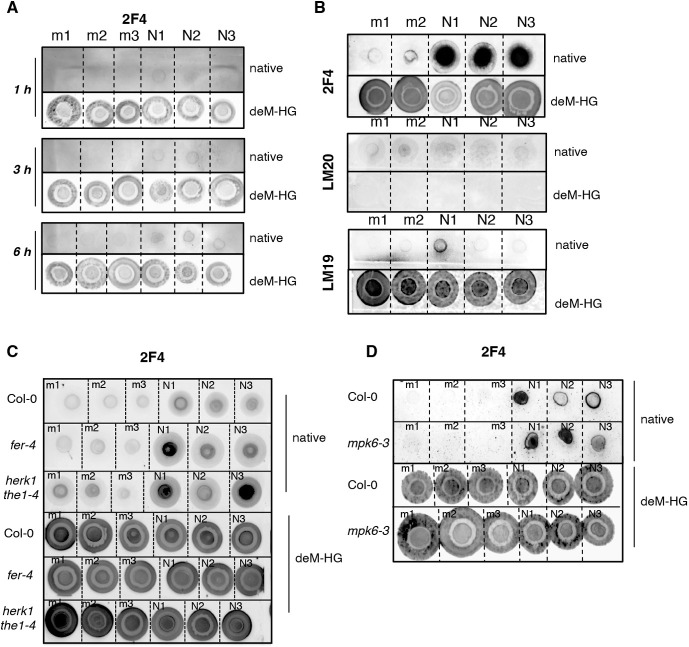
**Salt alters the degree of methylation in loosely bound pectins.** (A) Seven-day-old *Arabidopsis* Col-0 seedlings were treated for 1, 3 and 6 h with water (mock, ‘m’) or 100 mM NaCl (‘N’). From the AIRs, 5 μg sugars were extracted and treated with double-distilled water (native) or 0.1 M Na_2_CO_3_, pH 11, to obtain de-methylated HG (deM-HG). Dot blots was performed using 2F4 (*n*=3). (B) Five micrograms of sugars extracted from 6-day-old seedlings after 24 h treatment with water (m) or 100 mM NaCl (‘N’) were used to perform dot blots as in A. Results using the 2F4, LM20 and LM19 antibodies are shown (*n*=3). (C,D) Six-day-old seedlings of the Col-0, *fer-4* and *herk1 the1-4* (C) or Col-0 and *mpk6-3* (D) lines were treated as in B. Sugars were extracted and 5 μg spotted on nitrocellulose membranes after 1 h incubation with double-distilled water (native) or Na_2_CO_3_, pH 11 (deM-HG). Dot blots were performed using the 2F4 antibody (*n*=3).

CrRLK1Ls were previously reported to be essential in mediating the CWI-sensing maintenance mechanism. To investigate salt-induced CW modifications, we analyzed pectins isolated from mock- and salt-treated wt seedlings in a time-course experiment (Fig. 3A). The extracted CW material was subjected to pectin extraction by using boiling distilled water. Total sugars were quantified (Dubois et al., 1951; DuBois et al., 1956) and sugars were spotted onto nitrocellulose membrane to perform dot blot analyses. In all blots, the same amounts of sugars were used in the upper part of the dot blots (native pectins) as in the lower parts of the membranes, with the exception that the latter were treated with Na_2_CO_3_ at pH 11 to remove HG methyl esters, functioning as controls for the probes used.

Already after 1 h of salt treatment (Fig. 3A), we could detect enhanced signals using the CW probe 2F4, which recognizes partially de-methyl-esterified HG (deM-HG), suggesting that the salt-triggered CW modification occurs early in response to the stimulus. Interestingly, the signal increased over time and was the most distinct at 24 h (Fig. 3A,B), likely suggesting that pectin de-methyl esterification increases and/or spreads over tissues in salt-treated seedlings. The 2F4 probe, which was previously reported to specifically recognize Ca^2+^-crosslinked HG dimers (Douchiche et al., 2010; Liners et al., 1989), showed enhanced signals in the Na_2_CO_3_-treated extracted sugars (Fig. 3A-D, deM-HG panel), suggesting that this probe binds, in fact, partly or fully de-methyl-esterified Ca^2+^-accessible HG (as also reported by Peaucelle et al., 2008).

To examine pectin structural organization further, we used additional CW probes, including LM19 and LM20 (Fig. 3B), which recognize fully de-methyl-esterified and highly methyl-esterified HG, respectively (Peaucelle et al., 2008; Verhertbruggen et al., 2009), as well as LM18 and JIM5 (Fig. S3A), which, similar to 2F4, recognize partially methyl-esterified HG. When wt seedlings were treated with salt, we observed that water-extracted CW pectins displayed enhanced 2F4, LM18 and JIM5 signals (Fig. 3B; Fig. S3A) and slightly enhanced LM19 signal, whereas no differences were detected in LM20-incubated samples. These results clearly show that salt reduces the degree of methylation of homogalacturonan, as measured by different independent probes.

Interestingly, analysis of pectin methyl esterification in the salt-sensitive *fer-4* and *herk1 the1-4* mutants showed that, similarly to the wt, they also display changes in the overall methyl esterification distribution upon salt stress, suggesting that in these mutants, structural modifications of the CW were also induced by salt treatments, as observed in the wt (Fig. 3C; Fig. S3B). Comparable results were also obtained using *mpk6-3* mutant seedlings (Fig. 3D), suggesting that even if MPK6 seems essential to activate the signaling in response to perception of salt stress, it is not likely to be involved in regulating the synthesis and/or the enhanced CW de-methyl esterification detected after salt application.

### Inhibition of salt-induced pectin methyl esterase activation alleviates intracellular salt responses

**Fig. 4.**
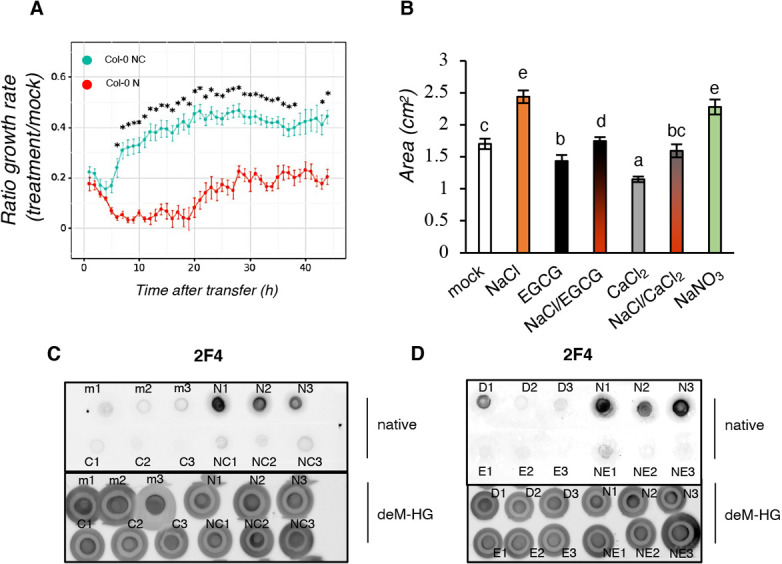
**Salt-induced PME activation is inhibited by CaCl_2_ and EGCG application.** (A) Root growth rate elongation in 7-day old Col-0 seedlings treated with mock, 125 mM NaCl (‘N’), 5 mM CaCl_2_ or a combination of NaCl/CaCl_2_ (‘NC’). Root growth rate elongation in 7-day-old Col-0 seedlings treated with mock, 125 mM NaCl, 5 mM CaCl_2_ or a combination of NaCl/CaCl_2_. Root growth rate is expressed as a ratio between NaCl and mock (‘N’) or NaCl/CaCl_2_ and mock (‘NC’). Each of the dots represent the average length of two independent experiments (*n*=30 seedlings per treatment). Statistical comparisons were performed by using a Levene's test, followed by two-tailed, unpaired *t*-test; *P<0.05. (B) PME activity analyzed in 5 μg protein extracts spotted on plates containing highly methyl-esterified pectins with water (mock), 100 mM NaCl, 50 μM EGCG, a combination of NaCl and EGCG (NaCl/EGCG), 5 mM CaCl_2_, a combination of NaCl and CaCl_2_ (NaCl/CaCl_2_), or 100 mM NaNO_3_. Error bars indicate s.d. *n*=3. One-way ANOVA and Tukey's HSD (α=0.05) were used to compare treatments. Different letters indicate statistical significance between groups. (C) Six-day-old *Arabidopsis* Col-0 were treated for 24 h with water (mock, ‘m’), 100 mM NaCl (‘N’), 5 mM CaCl_2_ (‘C’) or a combination of NaCl/CaCl_2_ (‘NC’) (*n*=3) or DMSO (‘D’), 100 mM NaCl (‘N’), 50 μM EGCG (‘E’) or a combination of NaCl/EGCG (‘NE’) (*n*=3). Five micrograms of total sugars were extracted and dot blots performed using the 2F4 antibody.

Given that alterations in HG methyl esterification patterns mostly depend on enhanced activity of PMEs, enzymes required for pectin de-methyl esterification, we analyzed whether salt could increase PME activity *in vitro* to corroborate the observations made *in vivo*. First, we measured the PME activity in protein extracts of salt-treated and mock-treated wt seedlings in a time course. As a control, we included a treatment with (−)-epigallocatechin gallate (hereafter EGCG), a chemical inhibitor of PME activity (Lewis et al., 2008; Wolf et al., 2014). No statistically significant difference was detected between timepoints or between treatments in terms of PME activity (Fig. S4B,C), nor in previously reported analyses of PME activity in pectin-containing plates or the kinetics of the release of methanol (Mueller et al., 2013), suggesting that salt does not act through changing the amount of extractable PMEs. To support this observation, we performed *in silico* analysis of the gene expression of PME-encoding genes (selected according to Wang et al., 2013) using publicly available transcriptomic profiling in response to salt stress in *Arabidopsis* (Kilian et al., 2007). These data show that salt only has a limited effect on *PME* transcription (thus unlikely influencing their protein abundance) (Fig. S4D), supporting the idea that salt does not change PME abundance but instead might directly alter PME activity. When directly supplemented in the plate assay (Fig. 4B), the inhibitory effect of ECGC as well as a strong induction in PME activity by Na^+^ ions (either in the form of NaCl or NaNO_3_ application) was detected. Because Ca^2+^ was reported to alleviate *fer-4*-dependent salt-induced root phenotypes (Feng et al., 2018), and because we observed that it was sufficient to mitigate the severe salt-dependent root quiescent phase in wt seedlings (Fig. 4A; Fig. S4A), we tested the effect of Ca^2+^ on PME activity. We found that exogenous Ca^2+^ not only inhibited PME activity, as it was previously reported in the fruits of tomato and raspberry plants (Sobral et al., 2021; Yan et al., 2022) but also alleviated the salt-triggered activation of PME *in vitro* (Fig. 4B). To confirm the inhibitory effect of CaCl_2_ on salt-induced pectin de-methyl esterification, we analyzed HG methylation patterns and discovered that when wt seedlings were treated with a combination of salt and calcium (Fig. 4C; Fig. S4E), or salt and EGCG (used as a control, Fig. 4D; Fig. S4E), 2F4 signals were attenuated, suggesting that salt-dependent de-methylation of HG leads to reduced CW strength, which can be alleviated by exogenous application of chemicals that directly regulate the function of enzymes required for pectin relaxation.

### Salt-induced gene expression and MPK6 phosphorylation are mitigated by addition of Ca^2+^ or EGCG

**Fig. 5.**
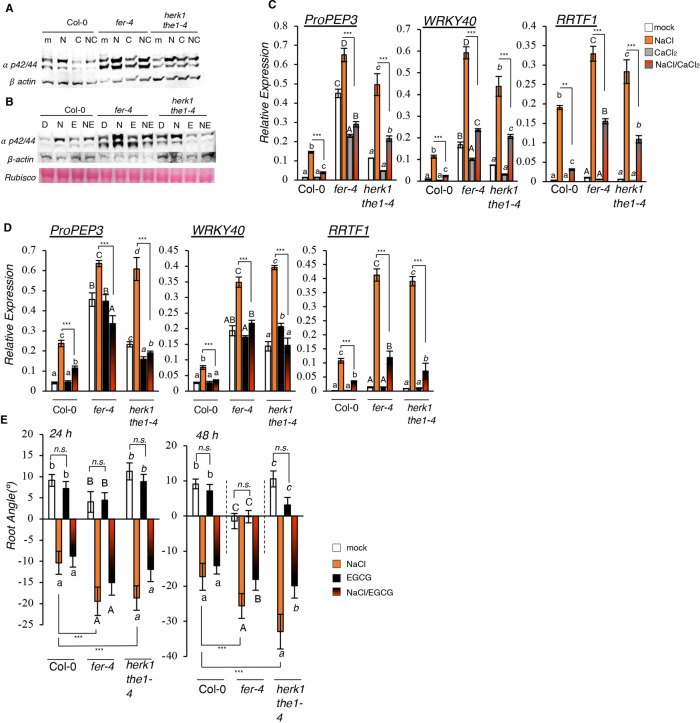
**Salt-induced responses are alleviated by CaCl_2_ or EGCG application.** (A,B) Seven-day-old seedlings of Col-0, *fer-4* and *herk1 the1-4* lines were treated for 15 min with water (mock, ‘m’), 100 mM NaCl (‘N’), 5 mM CaCl_2_ (‘C’) or NaCl/CaCl_2_ (‘NC’) (A) or with DMSO (‘D’), 100 mM NaCl (‘N’), 50 μM EGCG (‘E’) or NaCl/EGCG (‘NE’) (B). MPK phosphorylation was detected by immunoblotting. Equal loading was checked by using the anti-β-actin antibody. Similar results are shown in Fig. S5A,B. (C,D) Expression of *PROPEP3*, *WRKY40* and *RRTF1* determined by qRT-PCR was performed in seven-day-old seedlings of the Col-0, *fer-4*, *herk1 the1-4* lines treated for 1 h with water (mock, ‘m’), 100 mM NaCl (‘N’), 5 mM CaCl_2_ (‘C’) or NaCl/CaCl_2_ (‘NC’) (C) or with DMSO (‘D’), 100 mM NaCl (‘N’), 50 μM EGCG (‘E’) and NaCl/EGCG (‘NE’) (D). *n*=3. Error bars indicate standard deviation (s.d.). One-way ANOVA and Tukey's HSD (α=0.05) were used to analyze statistical differences within the same genotype (lowercase letters for Col-0, uppercase letters for *fer-4*, italic letters for *herk1 the1-4*). Multiple two-tailed, unpaired *t*-tests according to Benjamini–Hochberg correction between NaCl treatments and NaCl/CaCl_2_ treatments are shown. **P<0.01; ***P<0.001. (E) Four-day-old seedlings of the Col-0, *fer-4* and *herk1 the1-4* lines were subjected to the halotropism assay with DMSO (mock), 200 mM NaCl, 50 μM EGCG or NaCl/EGCG. Histograms represent the average root angle of three independent experiments, error bars show s.e.m. *n*=75. Different letters indicate statistical significance by one-way ANOVA and Tukey's HSD (α=0.05) within the same genotype (lowercase letters for Col-0, uppercase letters for *fer-4*, italic letters for *herk1 the1-4*). Asterisks indicate differences between salt treatments according to multiple two-tailed, unpaired *t*-tests coupled with Benjamini–Hochberg correction. ***P<0.001; n.s., not significant.

To investigate the cellular and physiological consequences of salt-induced HG de-methyl esterification, and to understand the extent to which the inhibition of salt-dependent PME activity through CaCl_2_ and EGCG application alter salt responses, we analyzed early and late stress responses in the presence of CaCl_2_ and EGCG. First, we tested the salt-dependent MPK6 phosphorylation and found that, as early as the 15 min timepoint, Ca^2+^ could alleviate salt-triggered MPK6 phosphorylation in the wt as well as in the salt-hypersensitive mutants *fer-4* and *herk1 the1-4* (Fig. 5A; Fig. S5A). Similar results were obtained when seedlings were treated with EGCG (Fig. 5B; Fig. S5B). In fact, we found that even if ECGC treatment alone did not affect MPK6 activation in any of the genotypes, the combined treatment with NaCl and EGCG reduced MPK6 activation (Fig. 5B; Fig. S5B), suggesting that the inhibition of salt-induced de-methyl esterification of pectin can alleviate the immediate stress responses that are reported in this study.

Next, we tested the effect of these chemicals in affecting salt-induced marker gene expression. When wt, *fer-4* and *herk1 the1-4* mutant lines were treated with a NaCl/CaCl_2_ combination, gene expression levels were inhibited to the same (*PROPEP3*, *WRKY40*) or almost similar (*RRTF1*) physiological expression levels, compared with the mock controls (NaCl/CaCl_2_ versus mock) (Fig. 5C). During the experiments performed with EGCG, we observed a trend similar to that seen in seedlings treated with NaCl/CaCl_2_ combinations. In fact, even if EGCG itself did not affect the expression of the selected markers, when EGCG was applied in combination with NaCl, gene expression induction levels were reduced most evidently in the salt-sensitive *fer-4* and *herk1 the1-4* mutants (Fig. 5D). To study the effect of EGCG on root halotropic response, we analyzed root direction (angle) for seedlings treated with NaCl on a gradient supplemented with or without EGCG. Mild but significantly reduced root bending was detectable in the *herk1 the1-4* and *fer-4* mutants, but not in Col-0 seedlings, after 48 h of co-treatment with NaCl/EGCG (Fig. 5E; biological replicates and root elongation rates are shown in Fig. S5C-E). EGCG itself did not alter root direction at any timepoint compared with the mock control (Fig. 5E, black versus white bars). However, as previously reported, Na^+^-dependent root halotropism was not mitigated by exogenous Ca^2+^ application (Fig. S5F,G; Galvan-Ampudia et al., 2013).

Taken together, these results suggest that salt-induced CW modification involves pectin architecture alterations caused by direct PME activation. We show that changes in pectin moieties occur quickly upon salt treatment, and that chemically mediated PME inhibition can partially counteract MPK6 phosphorylation and salt-dependent gene expression but not halotropism, which appears to be a salt-induced but CW-independent response. This hypothesis would also explain why salt-induced halotropism is not attenuated by Ca^2+^ application (Galvan-Ampudia et al., 2013). Moreover, our data show that the majority of the salt-induced responses are triggered after perception of salt-induced modification of pectin architecture and appear to partly require FER and/or HERK1/THE1, which could function in negatively regulating both salt-induced MPK6 phosphorylation and marker gene induction.

## DISCUSSION

In recent years, a mechanism required to monitor CW structure, namely CW integrity (CWI) maintenance, has been described in plants. In this mechanism, several proteins belonging to the CrRLK1L subfamily were described to function as CW sensors (Bacete et al., 2022; Engelsdorf et al., 2018; Gigli-Bisceglia et al., 2020; Vaahtera et al., 2019). In our attempt to understand the role of CrRLK1Ls in regulating salt stress responses, we studied the double *herk1 the1-4* mutant, which was originally reported to lack the function of both the CrRLK1Ls THE1 and HERK1, and only later characterized for being a combination of *herk1* loss-of-function and *the1-4* gain-of-function alleles (Merz et al., 2017). The double mutant shares stunted growth phenotypes similar to those of *fer-4* adult plants (Guo et al., 2009). We confirmed this observation and surprisingly discovered that these mutants also share a similarly enhanced salt sensitivity. We found that the *herk1 the1-4* double mutants showed more severe early responses to salt compared with the corresponding single mutants, suggesting that combinations between these two CrRLK1Ls alter salt responses (our findings) and non-salt-induced phenotypes (Guo et al., 2009). It is likely that PM-localized CrRLK1Ls (such as THE1, HERK1 and FER) regulate similar metabolic and cellular responses. For example, it has recently been reported that THE1, similarly to FER, functions as negative regulator of ABA synthesis in response to osmotic stress (Bacete et al., 2022; Zhao et al., 2021). Whether HERK1 together with THE1 would affect ABA metabolism and/or synthesis and would contribute to altering salt stress responses remains to be determined, but certain salt-induced FER-dependent phenotypes, such as stunted growth in salt, seem to be abolished upon disruption of ABA biosynthesis or ROS accumulation (Zhao et al., 2021).

Here, we have shown that the described phenotypic similarities between *herk1 the1-4* and *fer-4* do not depend on altered *FER* expression in the double mutant line (Fig. S1E), likely suggesting that impairment of FER function might not be the reason why *herk1 the1-4* has a *fer-*like response to salt. Potential transient hetero-oligomerization of different CrRLK1Ls has been reported, for example, in the case of HERK1/ANJEA or ANX/BUPS in pollen tubes (reviewed by Ge et al., 2019), and, thus, a possible interaction between HERK1 and THE1 cannot be excluded. Additionally, it is possible that HERK1/THE1 together alter FER activity, as FER has previously been reported to function as a scaffold protein in regulating the interaction with other PM-localized proteins that are responsible for modulating immune responses (Chen et al., 2016; Dünser et al., 2019; Stegmann et al., 2017).

Because *fer-4* mutants exhibit root-cell bursting in response to salt application (Feng et al., 2018), FER has been suggested to monitor CW pectin perturbations directly. In *Arabidopsis*, the CW pectins are rich in galacturonic acid and create HG strings. HG is transported to the CW in a highly methylated form and is de-methyl esterified in the apoplast by the action of PMEs (Lampugnani et al., 2018). Interestingly, FER's malectin domain has been found to preferentially bind pectin, with a specificity for de-methyl esterified pectin (Feng et al., 2018; Lin et al., 2022). To date, no available data demonstrate specific binding of other CrRLK1Ls to CW polymers; however, in yeast, in which the CWI mechanism was originally discovered, the extracellular domain of the WSC1 receptor can bind structural elements of yeast CWs, functioning as a sensor for the osmo-dependent CW/PM interphase modification (Dupres et al., 2009; Heinisch et al., 2010). In plants, THE1 has been hypothesized to function in a similar manner as the yeast WSC1, being required to activate responses induced by cellulose impairment-induced cell swelling. Recent evidence showed that, not only signaling, but also CW strength, might be altered in *the1* mutants (Bacete et al., 2022), suggesting that CrRLK1Ls might not only regulate the intracellular responses to CW damage, but also the structural architecture of the CW. Here, we show that changes in CW elasticity observed in response to salt (Feng et al., 2018) might be the result of modifications in the degree of methyl esterification of homogalacturonan, as wt seedlings treated with salt showed enhanced PME activity and increased de-methyl esterified epitopes of pectin compared with the mock controls (Fig. 3, Fig. 4A). It has previously been suggested that Na^+^ ions can intercalate in the de-methyl-esterified pectin, thus limiting Ca^2+^-mediated pectin crosslinks. De-methyl-esterified HG can be crosslinked by Ca^2+^, forming ‘egg-boxes’, pectin-crosslinked structures involved in promoting CW stiffening (Hocq et al., 2017). Consistent with this, excessive Ca^2+^ ions can counteract the effect of Na^+^, restoring the right balance of crosslinked pectins (Feng et al., 2018).

**Fig. 6.**
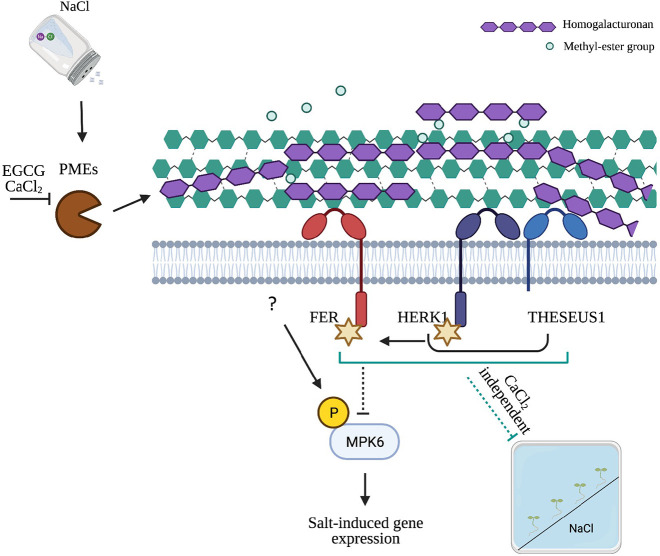
**Hypothetical model for cell wall modifications and CWI-induced signaling pathways triggered by salt stress.** Salt application directly modifies the activity of pectin methyl-esterase (Fig. 4B) triggering cell wall de-methyl esterification (Fig. 3). Detection of these cell wall modifications seems to be responsible, at least in part, for the activation of salt stress responses. We show that chemicals that can inhibit PME activity (Fig. 4B) and HG methyl-esterification (Fig. 4C,D) can also alleviate the responses to salt stress (Fig. 5A-D). We hypothesize that the cell wall sensors FERONIA (FER) or HERK1/THESEUS1 attenuate most of the salt-dependent phenotypes, being required to negatively regulate the salt-dependent MPK6 activation in response to salt stress (Fig. 1C-E). However, because in control conditions these mutants already displayed minor enhanced marker gene expression as well as MPK6 activity (Fig. 1C-E, Fig. 5A-D), we speculate that their functional impairment might cause cell wall modifications, and this damage can be directly or indirectly alleviated by the presence of certain chemicals (Fig. 4B-D). We show that CaCl_2_ application reduces basal gene expression levels and physiological MPK6 activity is altered in the CWI mutants (Fig. 5A,C), likely suggesting that the dual effect of MPK6 on strongly inhibiting PME activity (Fig. 4B) and/or cell wall crosslinks, might result in a faster and stronger alleviation of the salt stress phenotypes. On the other hand, treatment with chemicals (such as salt), which have an impact on the cell wall composition, lead to a stronger damage response, which correlates with a higher intensity of responses in the cell wall sensor mutants. In this scenario, one branch of the responses might depend on a direct inhibition mediated by the cell wall sensors of the downstream responses, whereas the other branch (represented by a question mark) might be uncoupled but still be dependent on their effect on the physiology of the cell walls. At the same time, the cell wall sensors seem to only mildly affect halotropism, a root-bending response that occurs independently of MPK6 and/or CaCl_2_ application. It should be noted that all the cell wall analyses reported in this paper derive from whole *Arabidopsis* seedling extracts and the tissue or cell types in which cell wall modifications occur in response to salt remain to be determined.

It has been reported that pectin modifications influence the strength of the CW as well as cell shapes (Pelloux et al., 2007; Rui and Dinneny, 2020). In pollen tubes, however, although lower HG esterification is associated with higher CW stiffening, several other studies show that the reduction of HG methyl esterification patterns enhance CW expansion (Haas et al., 2020; Peaucelle et al., 2011). Atomic force microscopy-based analyses of the indentation on shoot apical meristems of plants with reduced pectin methyl esterification showed enhanced CW softening (Haas et al., 2020; Peaucelle et al., 2011). It has been hypothesized that in the absence of Ca^2+^, de-methyl-esterified pectins display altered fluidity, being more susceptible to the action of pectin-degrading enzymes such as polygalacturonases (PGs), which enhance CW extensibility (Wakabayashi et al., 2003; Peaucelle et al., 2008; Wang et al., 2020). We now show that exogenous application of Ca^2+^ strongly inhibits the salt-dependent PME activation, and that this effect is also visible at the CW level (Fig. 4B), where the salt-induced HG demethylation is strongly suppressed by Ca^2+^ co-treatment (Fig. 4C). At the concentration we used, Ca^2+^ had a stronger effect compared with the PME-inhibitor EGCG in terms of its ability to inhibit PME activity *in vitro* (Fig. 4B). As observed for the wt seedlings, and also in the salt-treated *fer-4* and *herk1 the1-4* mutants, Ca^2+^ application reduced the amplitude of the gene marker induction, and interestingly, unlike EGCG, it reduced the basal gene expression levels and the physiological MPK6 activation detected in the CWI mutants. We hypothesize that the differences between CaCl_2_ and EGCG might be explained by the dual effects of Ca^2+^ ions on both PME activity regulation and CW crosslinks, resulting in the alleviation of salt-dependent and -independent CW defects. Pre-existing CW defects in *fer-4* and *herk1 the1-4* mutants might explain the differences in the intensity of the responses (e.g. the marker gene expression and MPK6 activity) in mock and in salt treatments. In this scenario, we hypothesize the presence of one signaling branch that is activated by the salt-dependent alteration of pectin structure, which requires the CW sensors FER or the combination of HERK1/THE1, in inhibiting salt-triggered responses (Fig. 6). The other signaling branch, represented in Fig. 6 with an arrow that goes from the cell membrane to MPK6, is dependent on salt-induced CW damage, but is likely uncoupled from the active function of the CW sensors while still being dependent on their effect on the physiology of the CWs. In this case, MPK6 activation seems to be partly independent of the presence of the CW sensors per se, but might be dependent on their role in indirectly altering CW composition, leading to the activation of CW damage responses, which can be alleviated by chemicals that reinforce the CWs.

Because we did not focus on the cellular CW modifications, we cannot conclude whether PME localization is altered in specific tissues of the *fer-4* and *herk1 the1-4* mutants. We have observed a similar increase of de-methyl-esterified pectins upon salt treatment when comparing mutants with wt (Fig. 3C), suggesting that the direct effect of salt on PME activity seems to be similar between the genotypes. However, as we analyzed pectin in whole seedlings, we cannot exclude the possibility that these results are the outcome of CW compensatory mechanisms. In other words, salt-induced PME activity might be enhanced in specific tissues when the CW sensors are missing, ultimately altering the overall downstream responses (gene expression and MPK6 phosphorylation). This would imply that PME activity is controlled by both salt and CrRLK1L presence. It would also be consistent with the observation that both Ca^2+^ and EGCG application could mitigate the stronger responses observed in salt-treated *fer-4* and *herk1 the1-4* mutants, likely inhibiting the enhanced PME activity. Recently, a role for another member of the CrRLK1Ls family, ERULUS, in controlling the methyl esterification of root hair CW has been reported, suggesting that CrRLK1L members might be required for controlling the functionality of CW enzymes, and not only for CW alterations (Schoenaers et al., 2018). Previous research has shown that PME activity increases with salt concentration and is altered by pH (Cameron et al., 2003; Hultin et al., 1966; Kent et al., 2016), yet the identification of the targeted PMEs and PME inhibitors (PMEIs) is far from being completely understood. Because of functional redundancy of PMEs and the the high number of family members, and because salt does not strongly alter PME transcription, finding hints on which PME or PMEIs in concert could specifically play a role in response to salt stress would require meticulous analysis. In the literature, examples of observations either contrasting or supporting our hypothesis can be found. For example, the positive function of PME31 in regulating salt stress responses is in contrast to our hypothesis that salt increases PME activity, possibly enhancing salt stress responses, as *pme31* mutant lines show hypersensitive phenotypes to salt stress, likely suggesting that PME31 impairment is detrimental for salt tolerance (Yan et al., 2018). By contrast, it has been reported that reduced expression of *PMEI10* (*AT1G62760*) leads to enhanced salt tolerance, likely suggesting that PMEI10 might function as negative regulator of salt stress responses (Jithesh et al., 2012; reviewed by Wormit and Usadel, 2018). Moreover, apart from identification of the specific PMEs, the effect that EGCG had on the halotropic root bending also deserves further study. Root halotropism has been reported to occur independent of extracellular Ca^2+^ (our findings and Galvan-Ampudia et al., 2013) and regulated by PIN-FORMED 2 (PIN2) re-localization (Galvan-Ampudia et al., 2013). Although halotropism seems to be independent of the function of MPK6 (Fig. S2C), the salt-sensitive *fer-4* and *herk1 the1-4* mutant lines showed a mild but enhanced salt-induced root bending after 48 h after salt application. Whether the slightly enhanced halotropic response in *fer-4* and *herk1 the1-4* could be due to mis-regulation of PIN2 is still unknown. In a recent paper, the altered root gravitropic response observed in *fer-4* has been associated with altered PIN2 re-localization (Dong et al., 2019), which might also explain our observations. In our experiments, EGCG could alleviate the enhanced halotropism observed in *fer-4* and *herk1 the1-4* mutant lines. Although the molecular mechanism is still unclear, it has been shown that EGCG application affects the localization and dynamics of several PM-localized proteins (including PIN3 and FLS2) (McKenna et al., 2019), likely suggesting that the ECGC-dependent alteration of protein dynamics could impair the functionality of proteins required for the halotropic stress responses.

In this paper, we demonstrate that salt directly induces PME activity, changing the composition of loosely bound pectins. We also discovered that pectin modification is an essential step for the activation of certain downstream responses, such as MPK6 phosphorylation, which is in turn the central hub required for the full activation of salt-induced marker gene expression. We report that CaCl_2_ or EGCG application alleviates salt responses in an MPK6-dependent manner, and that both chemicals function at the CW, inhibiting the salt-dependent PME activation and linking some of the salt-dependent responses to changes in pectin composition. The inhibition of MPK6 activation has been demonstrated previously to make plants more resistant to salt (Han et al., 2014). Accordingly, we show that MPK6 positively regulates salt-induced gene expression, which could affect salt tolerance. Because we could not explain why PME inhibition leads to a reduced salt response in the cell wall-sensing mutants, in our model (depicted in Fig. 6), we hypothesized the presence of a distinct branch that regulates MPK6 phosphorylation and gene expression in response to salt, independent of the cell wall sensors. However, lack of MPK6 or addition of Ca^2+^ did not affect the halotropic root-bending response, which seemed to be alleviated upon EGCG application. Further investigations will be required to understand the molecular mode of action of CrRLK1Ls in partly controlling responses to CW changes induced upon salt stress.

## MATERIALS AND METHODS

### Plant materials and growth conditions

*Arabidopsis thaliana* ecotype Col-0 was used as wild type. Experiments were conducted using several mutants listed in Table S1. Before sowing, seeds were surface sterilized with 50% bleach for 10 min and washed three times with sterile distilled Milli-Q water. The seeds were sown after 2 days of vernalization at 4°C in the dark. For MPK6 phosphorylation assays and gene expression analysis, 15 seeds were germinated and grown in 2 ml half-strength Murashige and Skoog (MS) medium supplemented with 0.1% 2-(N-morpholino)ethanesulphonic acid (MES) buffer and 0.5% sucrose (pH 5.7) in 12-well plates. The solutions used for the analysis of the effects of Ca^2+^ on salt-induced responses were distilled sterile Milli-Q water (used as mock), 5 mM CaCl_2_, 100 mM NaCl or a combination of 5 mM CaCl_2_ and 100 mM NaCl (referred throughout as NaCl/CaCl_2_). Similarly, the solutions used for the analysis of PME inhibition were dimethyl sulfoxide (DMSO; mock), 50 µM EGCG (Sigma-Aldrich; in DMSO), 100 mM NaCl or a combination of EGCG/NaCl. All these experiments were performed on 7-day-old seedlings. All treatments contained equal amounts of DMSO. For the halotropism assays, the media used were made with the recipe stated above and supplemented with 1% agar. Seedlings were grown in custom-made holders inclined at an angle of 70° and, after 4 days, the lower part of the media under a diagonal line (Galvan-Ampudia et al., 2013) was cut using a sterile, thin glass slide and replaced with 1/2 MS supplemented with water (mock) or 200 mM NaCl. In the case of halotropism assays performed in the presence of EGCG, the agar strength of the medium was enhanced to 1.25% to avoid root-tip growth into the medium. In these experiments, the lower gradients contained DMSO (mock), 50 µM EGCG, 200 mM NaCl or a combination of EGCG/NaCl. All treatments contained equal amounts of DMSO. All results are based on independent experiments and the numbers of biological replicates are stated in the figure legends or shown in supplemental figures. An Epson Scanner was used to image plates at 24 h or 48 h after treatment. For CW extraction analysis and ion content measurements, seedlings were grown for 6 days (in the case of 24 h treatment) or 7 days (for the time-course analysis) in 250 ml Erlenmeyer flasks containing 110 ml of 1/2 MS medium, 0.1% MES and 1% sucrose (pH 5.7) on a flask shaker at a constant speed of 130 rpm. Treatments were performed with water or 100 mM NaCl and samples taken after 24 h. For time-lapse growth analysis, seedlings were grown in 1/2 MS medium, 0.1% MES (pH 5.7) supplemented with 1% agar in square plates positioned in vertical holders (90°). After 7 days, seedlings were transferred in water- (mock) or 125 mM NaCl-containing plates and images taken using an automated system every 20 min for over 42 h. For the bleaching assays, seeds were germinated and grown for 10 days in horizontally oriented square plates containing 1/2 MS medium, 0.1% MES, 1% agar (with or without 150 mM NaCl), pH 5.7. Seedlings were grown in long-day conditions (16 h light, 22°C/8 h dark, 18°C) at 150 μmol m^−2^ s^−1^ photon-flux density.

### Salt stress assays

Analysis of root halotropic responses was performed after scanning seedlings grown in square plates with an Epson scanner. Roots were traced with SmartRoot 2.0, an open-source plugin for Fiji/ImageJ (https://fiji.sc) and root directions were used to determine the angle of root bending. For time-lapse-based root assays, an automated script based on root-edge detection was developed (https://github.com/jasperlamers/timelapse-backtracing) and used to detect and trace root length using the last image taken. For salt-tolerance assays, plants were grown in pots under short light photoperiods (8 h light/16 h dark) in growth chambers set at 21°C and with controlled humidity (70%). Ten days after germination (1-week-old seedlings), the plants were transferred to new pots saturated with water or 75 mM NaCl. For 3 weeks, the plants were watered twice a week with Milli-Q water. Rosette fresh weights were measured at the end of the 3 weeks. The experiments were repeated two times with similar results.

### Gene expression analysis and MPK6 phosphorylation assays

Seven-day-old seedlings, grown as stated above, were treated with mock, 100 mM NaCl, 5 mM CaCl_2_ or a combination of NaCl/CaCl_2_. Similarly, for the analysis of the effect of PME inhibition, seedlings were treated with DMSO (mock), 50 µM EGCG (Sigma-Aldrich; in DMSO), 100 mM NaCl or a combination of EGCG/NaCl. After 15 min (MPK6 assays) or 1 h (gene expression analysis), seedlings were flash-frozen in 2 ml tubes containing metal beads in liquid nitrogen and ground into a fine powder using a paint shaker. Total RNA was isolated using the EasyPrep Plant Total RNA Extraction Miniprep (Bioland). DNase treatment was performed as described by Gigli-Bisceglia et al. (2018b) and Savatin et al. (2014) and cDNA synthetized using the iScript cDNA Synthesis Kit (Bio-Rad) according to the manufacturer's instructions. Quantitative reverse transcription PCR (qRT-PCR) analysis was performed using the CFX96 Real-Time System (Bio-Rad) and the qPCRBIO SyGreen Blue Mix Lo-ROX (Sopachem) master mix and primers (Table S2). Gene expression analyses were performed as described by Engelsdorf et al. (2018) and Gigli-Bisceglia et al. (2018b) and are shown as relative expression to *ACT2*, used as a reference gene. For protein extraction, the following extraction buffer was used: 50 mM Tris-HCl pH 7.5, 200 mM NaCl, 1 mM EDTA, 10% (v/v) glycerol, 0.1% (v/v) Tween 20, 1 mM phenylmethylsulfonyl fluoride (PMSF), 1 mM diothiothreitol (DTT), 1× protease inhibitor cocktail (Sigma-Aldrich, P9599), 1× phosphatase inhibitor (Sigma-Aldrich, P2850). After 15 min incubation on ice, samples were centrifuged at 13,000 ***g*** at 4°C for 15 min. The supernatants were used for western blot analysis. Protein concentration was assayed using the Bradford assay (Bio-Rad) and 15 µg of total proteins were loaded on 10% SDS-PAGE gels. The samples were mixed with 4× loading buffer [240 mM Tris-HCl pH 6.8, 8% (w/v) SDS, 40% glycerol, 5% β-mercaptoethanol, 0.04% (w/v) Bromophenol Blue] and heated for 2 min at 95°C. Protein samples were separated and then transferred to the nitrocellulose membrane using the Trans-Blot Turbo Transfer System (Bio-Rad) at 25 V, 1.3 A for 7 min. After blocking with Tris-buffered saline containing 0.1% Tween 20 (TBST) containing 5% (w/v) bovine serum albumin (BSA; Sigma-Aldrich, A3608) for 1 h, the membrane was incubated overnight with the primary phospho-p44/42 MAPK (Erk1/2) (Thr202/Tyr204) antibody (1:2000, Cell Signaling Technology, 9101). Subsequently, the membrane was washed three times with TBST and then incubated with the secondary anti-rabbit IgG, HRP-linked antibody (1:6000, Cell Signaling Technology, 7074) for 1 h. After washing three times with TBST, the chemiluminescent reaction was initiated by adding Clarity Western ECL Substrate (Bio-Rad, 1705061) for 2 min and detected by using the ChemiDoc imaging system (Bio-Rad). For equal loading analysis, membranes were stripped using stripping solution (100 mM Tris-HCl at pH 6.8, 2% SDS, 100 mM β-mercaptoethanol) for 1 h, and blocked as described previously before incubation with the β-Actin (C4) antibody (1:2000, Santa Cruz Biotechnology, sc-47778) for 1 h. Detection of chemiluminescence was performed as mentioned above.

### Cell wall extraction and dot blot assays

After 1, 3, 6 or 24 h treatment (see figure legends), seedlings were snap frozen in liquid nitrogen and ground into a fine powder with a paint shaker. At least three biological replicates for each treatment were used. Cell wall material (alcohol-insoluble residue, AIR) was extracted as described by Gigli-Bisceglia et al. (2018a). After drying overnight, the AIRs were weighed and sterile distilled Milli-Q water (pH 7.2) was used to extract pectins. Approximately 2-3 mg of AIR was boiled with 250 µl water for 1 h. After a brief centrifugation (15 min, 13,000 ***g***), the supernatants were collected and samples boiled again for 1 h with 250 µl water. After 1 h, the supernatants derived from the same samples were pooled. Total sugar content was measured using the phenol/H_2_SO_4_-based method using galacturonic acid as the standard (Dubois et al., 1951). Prior to spotting on the nitrocellulose membrane, 5 µg of total sugars were adjusted to 5 µl by adding (if necessary) distilled Milli-Q water (pH 7.2). For positive and negative controls, the same amounts of sugars were incubated for 1 h with NaCO_3_, pH 11, to remove pectin methyl esterification. Membranes were dried overnight at room temperature. Blocking [5% milk in phosphate-buffered saline (PBS)] was performed for 1 h. Next, the membranes were incubated for 1 h with the following primary antibodies: JIM5 (1:250 in 5% milk powder in 1×PBS), LM18, LM19, LM20 (1:250 in 5% milk in 20 mM Tris-HCl pH 8.2, 0.5 mM CaCl_2_, 150 mM NaCl) and 2F4 [1:250 in T/Ca/S buffer: Tris-HCl 20 mM (pH 8.2), CaCl_2_ 0.5 mM and NaCl 150 mM] (a gift from Prof. Paul Knox, University of Leeds, UK; PlantProbes) (Verhertbruggen et al., 2009). After three washes in PBS containing 0.1% Tween 20 (PBST), the membranes were incubated with the HRP-conjugated secondary antibodies against rat IgM (Agrisera, AS11 1224; 1:6000) for JIM5, LM18, LM19 and LM20 and against mouse IgG (Agrisera, AS11 1772; 1:6000) for 2F4 in 5% milk in PBS. After washing, chemiluminescence induced using the Clarity Western ECL Substrate (Bio-Rad, 1705061) and was detected with ChemiDoc imaging system (Bio-Rad).

### PME activity assay

*Arabidopsis* seedlings were grown for 7 days in 250 ml Erlenmeyer flasks containing 110 ml of 1/2 MS medium, 0.1% MES and 1% sucrose (pH 5.7) on a flask shaker at 130 rpm. Proteins were extracted from three biological replicates as described by Mueller et al. (2013) with minor modifications: NaCl was substituted with 500 mM KCl in the extraction buffer. Protein concentration was assayed using the Bradford assay (Bio-Rad) and 5 µg of total proteins were loaded on 10 cm Petri dishes containing 13 ml of 0.1% pectin that was 85% esterified (Sigma-Aldrich, P9561), 1% agarose, 12.5 mM citric acid, 50 mM Na_2_HPO_4_, pH 7.0, as described by Kohorn et al. (2014). Plates were supplemented with sterile water as mock (v/v), 100 mM NaCl, 5 mM CaCl_2_, a combination of NaCl/CaCl_2_, 50 µM EGCG, a combination of EGCG/NaCl, and 100 mM NaCO_3_. Staining and PME activity analysis were performed after incubation overnight at 37°C as previously described (Kohorn et al., 2014). For the experiments showed in Fig. S4B, 7-day-old, flask-grown seedlings were treated for 3, 6 or 24 h with mock, 100 mM NaCl, 50 µM EGCG or a combination of NaCl/EGCG. Five micrograms of protein extracts, obtained as described above, were loaded on pectin-containing Petri dishes and stained as described by Kohorn et al. (2014). The area of staining (in cm^2^) was analyzed using Fiji/ImageJ. For the PME activity, plate assay was performed according to Mueller et al. (2013), in which extracts were obtained from 7-day-old wild-type seedlings treated with mock, 100 mM NaCl, 50 mM EGCG or a combination of NaCl and EGCG (NaCl/EGCG), as in Fig. S4B.

### *In silico* PME expression analysis

The microarray dataset derived from Kilian et al. (2007) was used to determine the expression of genes encoding PMEs (as classified in Wang et al., 2013). From the dataset, salt-treated and control root samples we selected. *limma* software (Ritchie et al., 2015) was used to pre-process the data and determine the log fold change between salt and control samples at different timepoints (0.5, 1, 3, 6, 12 and 24 h). Genes that were not present on the ATH1 microarray chip were excluded from the analysis. The package pheatmap (version 1.0.12) was used to obtain the heatmap.

### Statistical analysis

IBM SPSS Statistics v24 was used to perform statistical significance (two-tailed, unpaired Student's *t*-test, ANOVA). Statistically significant differences are indicated with different letters for the one-way ANOVA/Tukey's HSD test at α=0.05 as reported in the figure legends. R (4.1.1) was used to perform statistical significance through pairwise *t*-testing with Benjamini–Hochberg corrections for multiple testing. Pairwise comparisons and statistically significant differences according to multiple testing coupled with Benjamini–Hochberg corrections are represented as *P<0.05, **P<0.01 and ***P<0.001.

For root time-lapse growth analysis, root elongation rate was calculated over 2 h intervals, averaged per hour and expressed either as a ratio between treatment and controls (Fig. 2A, Fig. 4A) or as growth rate (cm/h) (Fig. S4A). Each curve comprises several dots, representing the average growth rate and the corresponding standard error analyzed in each of the time-lapse images containing five seedlings per plates (only correctly segmented roots were included, *n*>12). In this assay, statistical comparisons were performed by analyzing every timepoint for equal variance using a Levene's test, followed by two-tailed, unpaired *t*-test to compare different genotypes at the same timepoint. The significance level (*P*=0.05) was corrected for multiple testing using stepwise Sidak correction.

## Supplementary Material

Supplementary information

Reviewer comments
