# Supplementary material for: Arabidopsis root responses to salinity depend on pectin modification and cell wall sensing
Source: Development. 2022 Jun 17;149(12):dev200363. doi: 10.1242/dev.200363 (PMC9270968; doi:10.1242/dev.200363)
Supplement: Supplementary information [file develop-149-200363-s1.pdf]

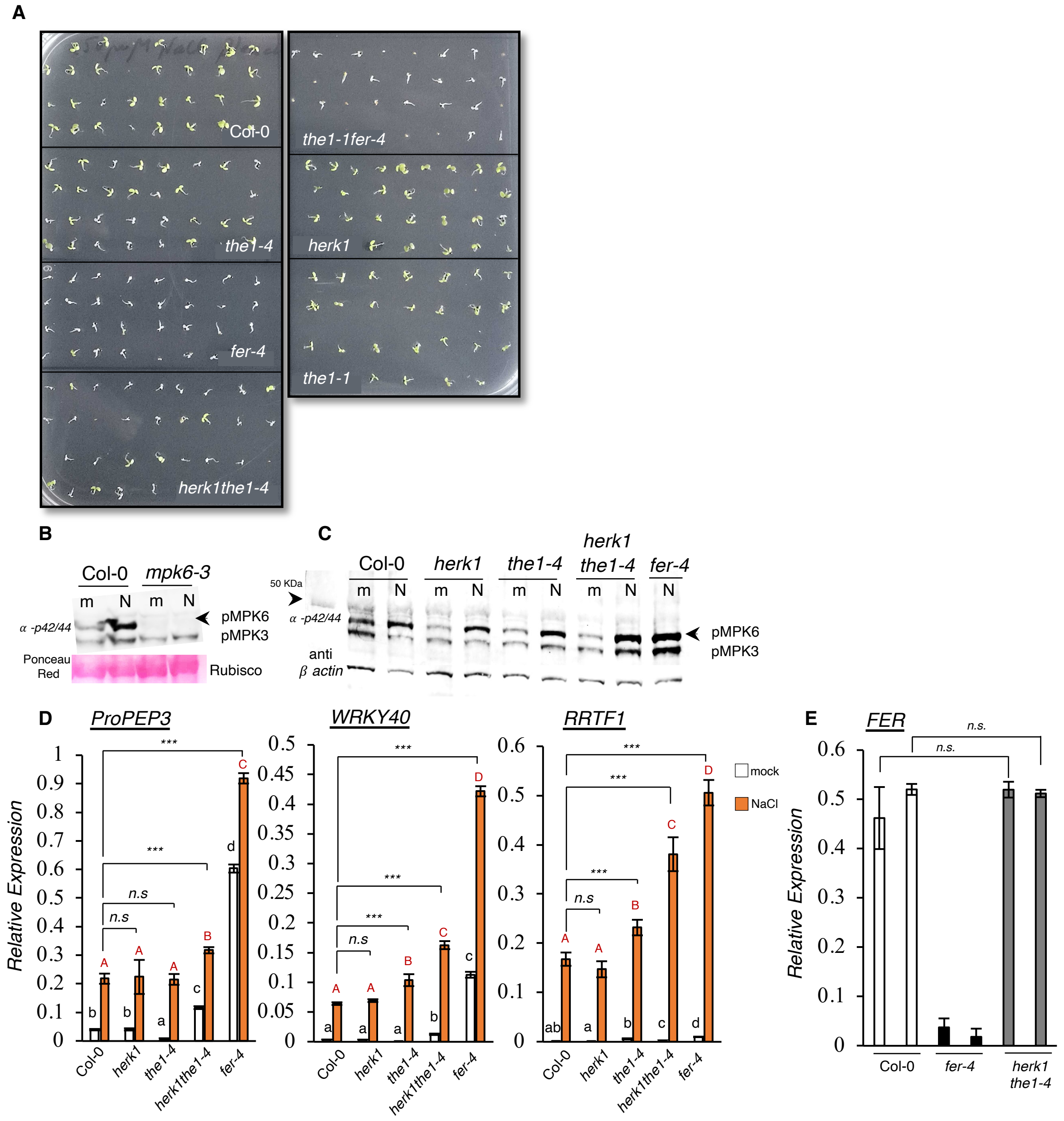

Fig. S1. A. Seeds of Col-0, *the1-1, the1-4, herk1, herk1the1-4, fer-4* and *the1-1fer-4* were germinated and grown for 10 days in 0.5% MS agar plates containing 150 mM NaCl. Similar results have been obtained in 4 different biological replicates. 7-day old seedlings grown in liquid 0.5% MS medium supplemented with 0.5% sucrose were treated for 15 minutes with H20 (mock, m) or 100 mM NaCl (N). MPK6 phosphorylation was detected in Col-0, *mpk6-3* **(B)** and in Col-0, *herk1, the1-4, herk1the1-4* and *fer-4* (**C**) by immunoblot using specific antibody against the phosphorylated forms of both MPK3 and MPK6 (α-*p44/42*). Equal loading is indicated with Ponceau Red staining or upon stripping on the same membrane by using β-actin Arabidopsis specific antibody. **D.** Seedlings grown as in B/C were treated for 1 h with H20 (mock) or 100 mM NaCl (NaCl) to analyze the expression levels of selected salt-induced marker genes. *ProPEP3, WRKY40, RRTF1* expression relative to *ACT2* was determined by qRT-PCR. Values represent means with error bars indicating SD. One-way ANOVA and Tukey's HSD (α= 0.05) was performed to compare different genotypes in the same treatment (black lowercase letters= mock, red uppercase letters= salt). n= 3. Asterisks show statistical comparisons between NaCl-treated Col-0 and *herk1the1-4* or *fer4* according to multiple paired T-tests and B-H correction. ***, P < 0.001, (n.s.= not-significant). **E.** Expression of *FER* was determined by qRT-PCR in 7-day old seedlings of Col-0, *fer-4* and *herk1the1-4* and represented as the average of 2 biological replicates (n= 6). Bars indicate standard deviation (SD). Asterisks show statistical comparisons between Col-0 and *herk1the1-4* according to multiple paired T-tests and B-H correction. (n.s.= not-significant).

Development • Supplementary information

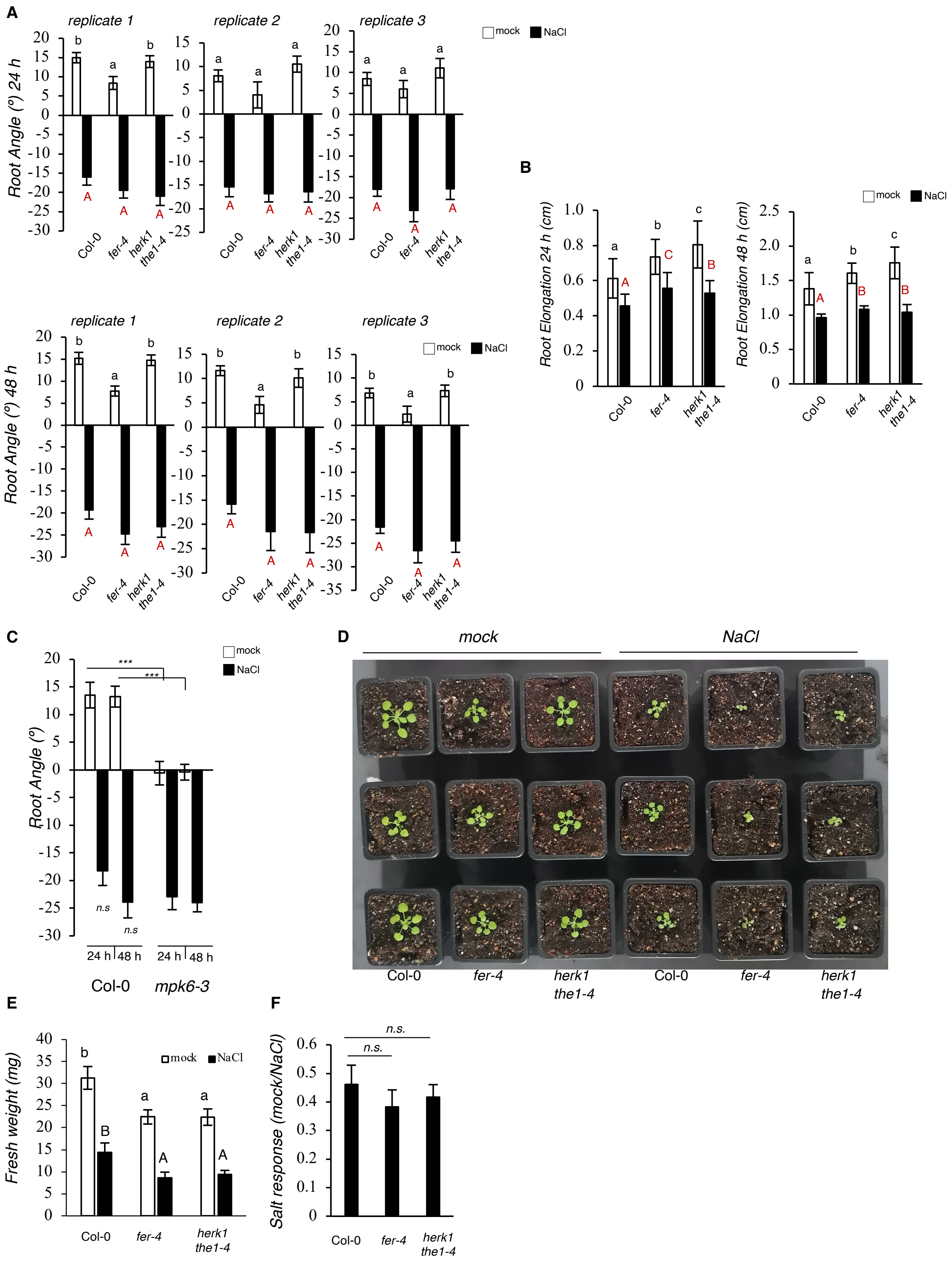
A
replicate 1
replicate 2
replicate 3
mock
NaCl
Root Angle (°) 24 h
Root Angle (°) 48 h
Col-0
fer-4
herk1 the1-4
B
Root Elongation 24 h (cm)
Root Elongation 48 h (cm)
C
Root Angle (°)
n.s
24 h
48 h
Col-0
mpk6-3
D
mock
NaCl
Col-0
fer-4
herk1 the1-4
E
Fresh weight (mg)
F
Salt response (mock/NaCl)
n.s.

Fig. S2. A. The 3 biological replicates pooled in figure 2B are shown (24 h, upper panel, 48 h lower panel). Bars indicating standard errors (SE). Letters indicate statistically significant differences according to One-way ANOVA and Tukey's HSD (α= 0.05) between genotypes within one treatment condition (n= 50) (black lowercase letters= mock, red uppercase letters= NaCl). **B.** Root elongation analyzed 24 h and 48 h after mock or NaCl gradient application in experiment presented in figure 2B. Bars indicate standard deviation. Letters indicate statistically significant differences according to One-way ANOVA in 3 independent experiments and Tukey's HSD (α= 0.05) between genotypes (black lowercase letters= mock, red uppercase letters= NaCl). **C.** 4-day old seedlings of Col-0, *mpk6-3* were treated as in figure 2B and root angle was analyzed after 24 h and 48 h. Histogram represents the average root angle of 2 independent experiments, while bars show the standard errors (SE). (n= 45). Asterisks show statistical comparisons between the same treatment in different genotypes according to Student's t-test. ***, P < 0.001, *n.s.* not significant. **D.** Representative rosette size of 4-week-old Col-0, *fer-4, herk1the1-4* plants grown in short day conditions and watered once with either MilliQ water (mock) or 75 mM NaCl (NaCl). Plants were photographed after 3 weeks of MilliQ watering. Rosette fresh weight reported in mg (**E**) and salt response (**F**) are shown. In E, values show means with error bars indicating standard errors (SE). Letters indicate statistically significant differences according to One-way ANOVA and Tukey's HSD (α= 0.05) between genotypes within one treatment condition (lowercase letters= mock, uppercase letters= NaCl). The experiment has been repeated twice with similar results. In F, bars represent a ratio between NaCl treated genotypes and the corresponding genotype in control conditions. Error bars are SE. *n.s.* = not significant according to one-way ANOVA in 2 independent experiments and Tukey's HSD (α= 0.05)

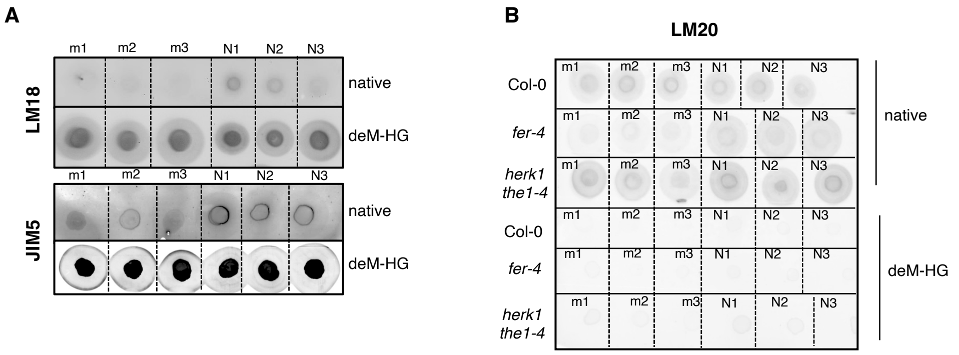

Fig. S3. A. 5 μg total sugars derived from Col-0 treated for 24 h with H20 (m) or NaCl (N) (Figure 3B) were spotted on nitrocellulose membranes after 1 h incubation with ddH2O (native) or Na2CO3 pH 11 (deM-HG). Dot-blot was performed with LM18 or JIM5. n=3. **B.** 5 μg total sugars derived from Col-0, *fer-4* and *herk1the1-4* (Figure 3C) were spotted on nitrocellulose and dot-blot was performed with LM20. Three biological replicates are shown.

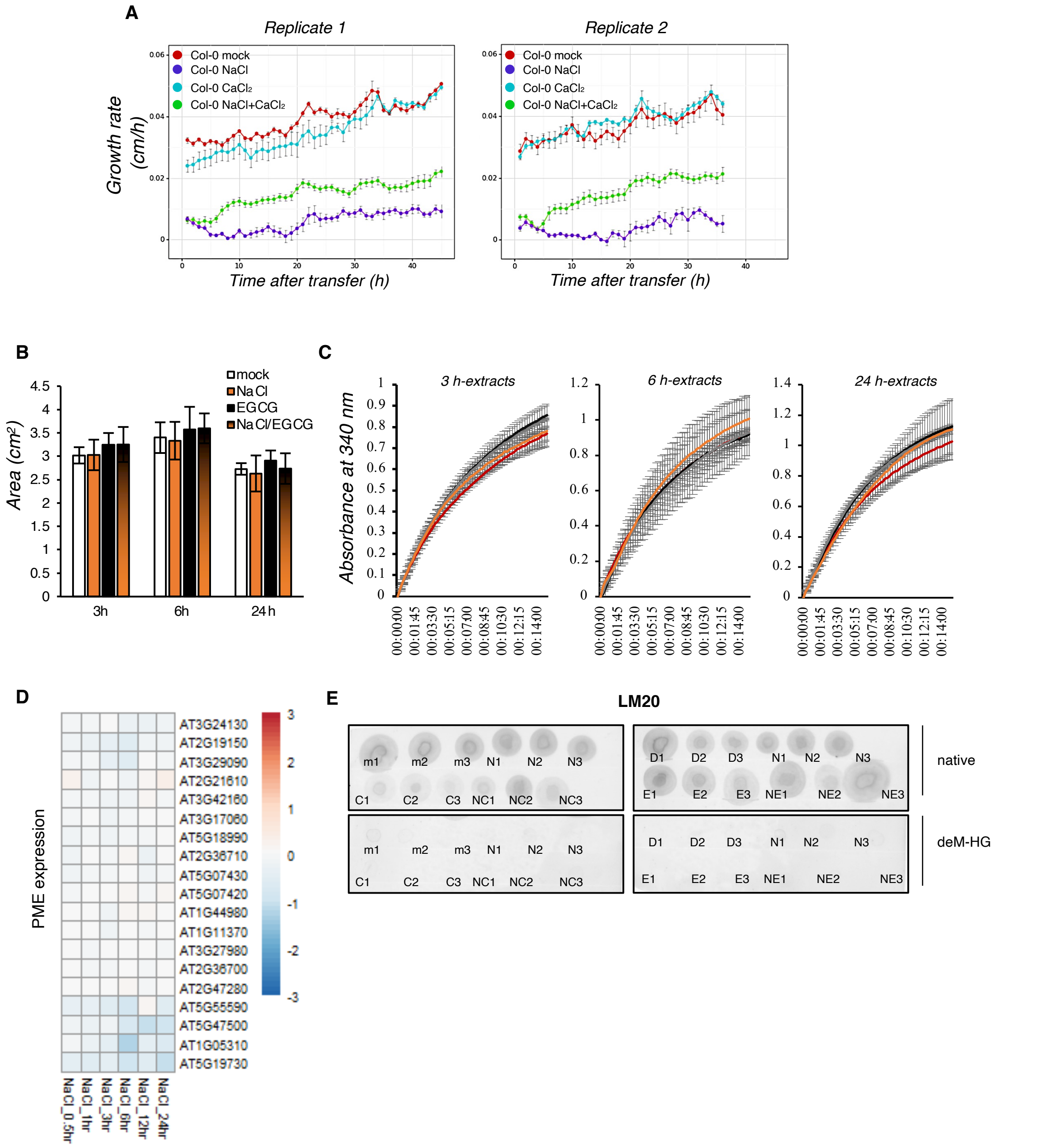

Fig. S4. A. Root elongation rates (expressed in cm/h) of the two independent biological replicates pooled in figure 4A are calculated as the average elongation over 2 h intervals in a 42 h time course experiment. Dots represent average elongation lengths and corresponding standard errors (n= 15 per treatment, per replicate). **B.** PME activity analyzed in 10 μg proteins extracted from wt seedlings treated for 3, 6 or 24 h with mock, 100 mM NaCl, 50 μM EGCG, a combination of NaCl and EGCG (NaCl/EGCG). Histogram indicates the average of 2 independent experiments. Bars are their standard deviation (SD) n=2. **C.** In plate assay PME activity performed in 10 ug protein extracts derived from 7-day old seedlings treated for 3, 6 or 24 with mock, 100 mM NaCl, 50 μM EGCG, a combination of NaCl and EGCG (NaCl/EGCG). Absorbance at 340 nm for each treatment is the average of 2 independent replicates consisting of 25 seedlings each. Bars show standard deviation. **D.** Expression of PMEs (as classified in Wang et al., 2013) was analyzed during salt stress by using public dataset (Killian et al., 2007). Plants were grown hydroponically and treated 18 days after sowing with 150 mM NaCl. Fold log changes between salt stress and control at 0.5, 1, 3, 6, 12 and 24 hours after salt stress are shown in a heatmap. **E.** 5 μg total sugars derived from Col-0 experiments presented in Figure 4C (left) or 4D (right) were spotted on nitrocellulose membranes after 1 h incubation with ddH2O (native) or Na_2CO_3 pH 11 (deM-HG). Dot-blot was performed with LM20. Three biological replicates are shown.

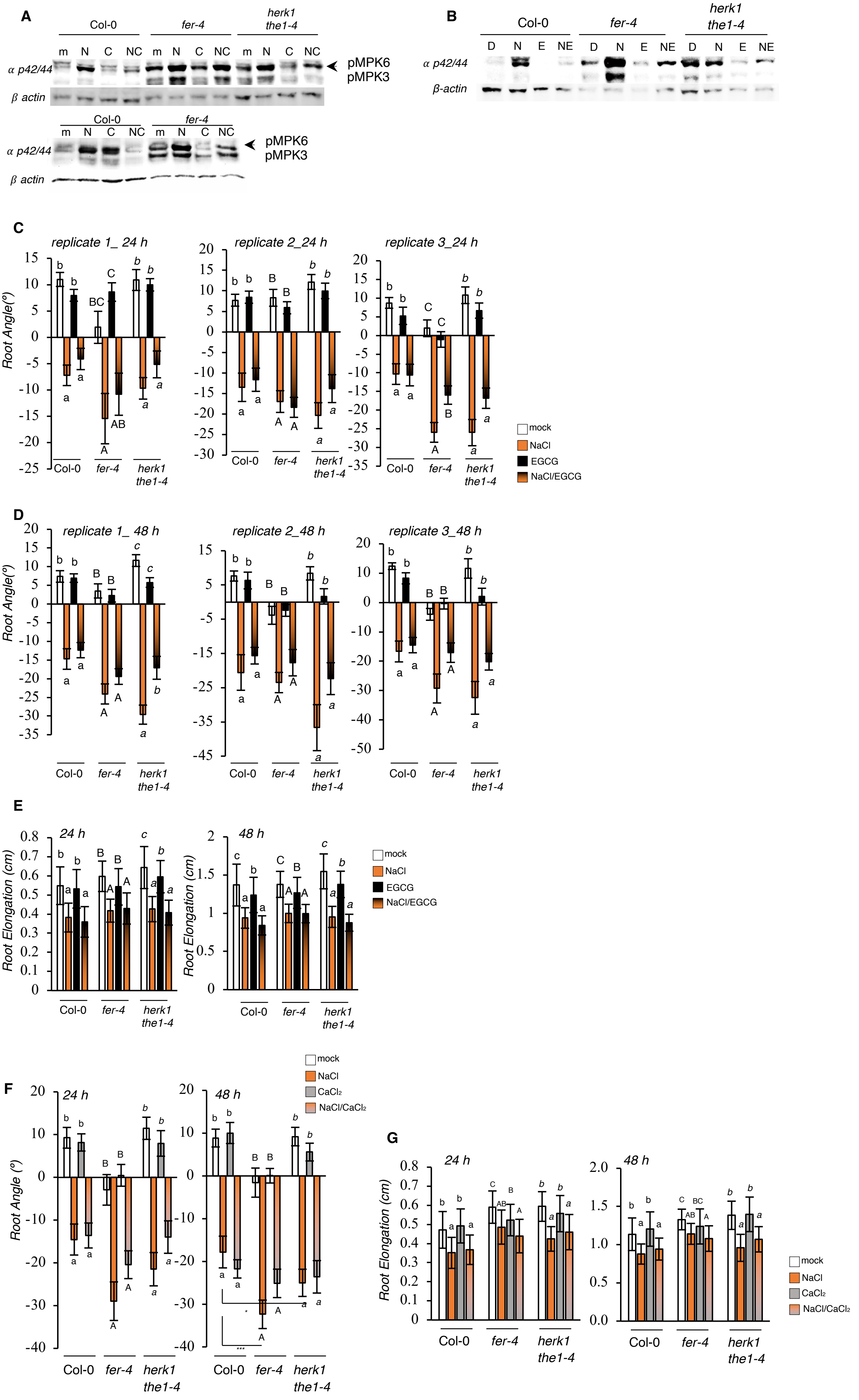

Development • Supplementary information

Fig. S5. A/B. Biological replicates of the experiment shown in figure 5A/B. **C/D.** Biological replicates of Figure 5E. Root angles analyzed at 24 h **(C)** or at 48 h (**D)** are plotted as average direction of 25 seedlings each treatment. Bars show standard error (SE). One-way ANOVA and Tukey's HSD (α= 0.05) was used to analyze statistical differences between treatments within the same genotype (lowercase letters= Col-0, uppercase letters= *fer-4*, italic letters=*herk1the1-4*). **E**. Root elongation of experiments shown in Figure 5E, analyzed after 24 h (left) and 48 h (right). Histograms represent the average root length of 3 independent experiments shown in C and D, while bars show standard deviation (SD). Statistical analysis has been performed as in C. (n= 75). **F.** 4-day old seedlings of Col-0, *fer-4* and *herk1the1-4* were subjected to mock, 200 mM NaCl, 10 mM $CaCl_2$ or NaCl/ $CaCl_2$ gradients. Root angle (direction) at 24 h and 48 h is shown. Histogram represents the average root angle of 2 independent experiments, while bars show the average of standard errors (SE). One-way ANOVA and Tukey's HSD (α= 0.05) was used to analyze statistical differences between treatments within the same genotypes (lowercase letters= Col-0, uppercase letters= *fer-4*, italic letters=*herk1the1-4*). Asterisks show statistical comparisons between the same treatment in different genotypes according to multiple paired T-tests according to B-H correction; ***, P < 0.001, *, P< 0.05 (n=45). **G.** Root elongation analyzed after 24 h and 48 h experiment shown in F. Histogram represents the average root length of 2 independent experiments, while bars show standard deviation (SD). Statistical analysis has been performed as in F. (n= 45).

Development • Supplementary information

Table S1. Arabidopsis genotypes used in this study.

Genotype	AGI	Background	Reference
the1-1	*AT5G54380*	Col-0	(Hématy et al., 2007)
the1-4	*AT5G54380*	Col-0	(Guo et al., 2009)
herk1	*AT3G46290*	Col-0	(Guo et al., 2009)
fer-4	*AT3G51550*	Col-0	(Duan et al., 2010)
the1-1 fer-4	*AT5G54380, AT3G51550*	Col-0	(Gonneau et al., 2018)
mpk6-3	*AT2G43790*	Col-0	(Liu and Zhang, 2004)
herk1the1-4	*AT5G54380, AT3G46290*	Col-0	(Guo et al., 2009)

Table S2. Primers used in this study.

Name	AGI	sequence (5'-3')	Reference
**ACT2_for**	*AT3G18780*	CTTGCACCAAGCAGCATGAA	(Czechowski et al., 2005)
**ACT2_rev**		CCGATCCAGACACTGTACTTCCTT	
**PROPEP3_for**	*AT5G64905*	CAACGATGGAGAATCTCAGA	(Engelsdorf et al., 2018; Gigli-Bisceglia et al., 2018)
**PROPEP3_rev**		CTAATTGTGTTTGCCTCCTTT	
**WRKY40 _for**	*AT1G80840*	GATCCACCGACAAGTGCTTT	(Denoux et al., 2008)
**WRKY40_rev**		AGGGCTGATTTGATCCCTCT	
**RRTF1-for**	*AT4G34410*	TATAGGAGCAAAGGCAAGTGCA	This manuscript
**RRTF1_rev**		ACTCCTCCATATTGCAATCCCC	
**FER_for**	*AT3G51550*	CGGAAAAGGAGTATGCGGTG	This manuscript
**FER_rev**		CTCCTACCACCGATGCTCAT	

Development • Supplementary information
