# Supplementary material for: Arabidopsis root responses to salinity depend on pectin modification and cell wall sensing
Source: Development. 2022 Jun 17;149(12):dev200363. doi: 10.1242/dev.200363 (PMC9270968; doi:10.1242/dev.200363)
Supplement: Reviewer comments [file develop-149-200363_review_history.pdf]

Arabidopsis root responses to salinity depend on pectin modification and cell wall sensing

Nora Gigli-Bisceglia, Eva van Zelm, Wenying Huo, Jasper Lamers and Christa Testerink
DOI: 10.1242/dev.200363

Editor: Yka Helariutta

Review timeline

Original submission:	21 April 2021
Editorial decision:	24 May 2021
Resubmission:	23 November 2021
Editorial decision:	26 January 2022
First revision received:	30 March 2022
Accepted:	29 April 2022

Original submission decision letter

MS ID#: DEVELOP/2021/199724

MS TITLE: Arabidopsis root responses to salinity depend on pectin modification and cell wall sensing

AUTHORS: Nora Gigli-Bisceglia, Eva van Zelm, Wenying Huo, Jasper Lamers, and Christa Testerink
ARTICLE TYPE: Research Article

Dear Dr. Testerink,

I have now received all the referees reports on the above manuscript, and have reached a decision. The referees' comments are appended below, or you can access them online: please go to BenchPress and click on the 'Manuscripts with Decisions' queue in the Author Area.

As you will see from their reports, the referees recognise the potential of your work, but they also raise significant concerns about it. Given the nature of these concerns, I am afraid I have little choice other than to reject the paper at this stage.

However, having evaluated the paper, I do recognise the potential importance of this work. I would therefore be prepared to consider as a new submission an extension of this study that contains new experiments, data and discussions and that address fully the major concerns of the referees. The work required goes beyond a standard revision of the paper. Please bear in mind that the referees (who may be different from the present reviewers) will assess the novelty of your work in the context of all previous publications, including those published between now and the time of resubmission.

Yours sincerely,

Yka Helariutta

Handling Editor
Development

© 2022. Published by The Company of Biologists under the terms of the Creative Commons Attribution License (https://creativecommons.org/licenses/by/4.0/).

Reviewer 1

Advance summary and potential significance to field

The authors of "Arabidopsis root responses to salinity depend on pectin modification and cell wall sensing" address how CrRLKs mediate salt response in Arabidopsis thaliana. With this work, the authors address a very relevant biological question of how plants deal with salinity which is generally detrimental for plant growth and development. The authors use a combination of a CrRLK loss (herk1) and a CrRLK gain-of-function mutant (the1-4) and characterize this mutant combination using several molecular approaches. Even though I believe that this project is very interesting for the field of plant biology, it is far from ready to be published. I encourage the authors to go on with this interesting project and publish it when it is in a more advanced state.

This manuscript suffers from the fact that it is very difficult to interpret the herk1/the1-4 mutant phenotype since it combines a gain and loss of function mutant allele from 2 genes from the same gene family. The authors show that the herk1/the1-4 resembles the phenotype of the CrRLK loss of function mutant fer-4. However, they to not manage to explain why a loss of function mutant of HERK1 and a gain of function mutant of THE1 displays in part similar phenoytpes like fer-4.

Comments for the author

The authors indicate that salt increases PME activity important for the growth adaptation to salinity. To support this hypothesis, the authors could check how PME overexpressing seedlings and pme loss of function mutants grow on salt. According to their data, one would expect that PME overexpressing seedlings show a partial salt resistance.
Another important question to address is whether MPK6 is required for salt triggered pectin methyl esterification. Previous work shows that CrRLKs also impact on pectin composition (for example schoenaers et al., 2018, current biology) suggesting that also MPK6 may have an impact on pectin composition.
It is in addition also very important that the authors improve their statistical analysis. Experiments with 2 variables (For example figure 1D and E) require a 2 way ANOVA test with an appropriate post hoc test (Tukey or Bonferroni). 1 way anova tests (as in figure 1D) and T tests (as in figure 1E) are not allowed. The authors should improve their statistical analysis throughout the whole manuscript.

Smaller issues.
- Some sentences in the manuscript are difficult to read. They would benefit from rephrasing. (for example p3 on the bottom: In our attempt to expand our current knowledge……….)
- The authors very often mention the HERK1/THE1-4 combination. However, the THESEUS gene is called THE1 and not THE1-4.

Reviewer 2

Advance summary and potential significance to field

The study by Gigli-Bisceglia and colleaugues sheds light on the intriguing question how cell wall pectins are involved in responses to salinity. It has been previously reported (Feng et al,, 2018) that the CrRLK1 FER is required for the recovery of root growth under salt stress after an initial growth reduction.
Here, the authors show that the combination of a HERK loss-of-function allele and a THE gain-of-function allele (both also CrRLK1s) show similar salt hypersensitivity as FER loss of function mutants. In addition, the authors show exaggerated responses to salt in herk1the1-4 and fer-4 mutants on the level of MAPK6 activation (minutes after NaCl) treatment, salt responsive gene expression (1 hour after treatment). cell wall composition (1 day after treatment) as well as growth responses (hours-days after treatment) such as growth rate and halotropism. Furthermore, the authors describe an alleviating effect of Calcium ions and the PME inhibitor EGCG on some of the responses and provide an alternative model to the study of Feng et al, implicating salt-induced changes in enzyme activity (e.g that of PMEs) as the factor triggering salt responses.
Interestingly, this study shows a calcium effect on signalling, indicating that the observed rescue is not only due to structural reinforcement. All of these experiments seem technically sound the

© 2022. Published by The Company of Biologists under the terms of the Creative Commons Attribution License (https://creativecommons.org/licenses/by/4.0/).

results are convincing, but their interpretation is very difficult (see below), and therefore, at this stage, i don't think they sufficiently support the model.

Comments for the author

Specific points:

-The genetic interactions are not addressed. THE1-4 is gain-of function allele, hence one would naively assume THE acts opposite to FER. The fact that herk1the1 have the same phenotype as fer-4 would formally suggest parallel pathways, thus the simplest explanation would be that FER and HERK are somewhat redundant and that the THE GOF counteracts FER enough to enhance the weak herk phenotype enough to end up in the range of fer loss of function. There might be other possibilities but they should be discussed.

-In addition to other issues with the model, MPKs can't be just simply downstream of CrRLKs, otherwise the various effects, including that of EGCG, in the absence of FER of HERK1 would be very difficult to explain.

-The difference in cell wall composition are convincing but their interpretation is very difficult. It is unclear when the cell wall changes happen, and they could very well represent a mitigation effort (to overcome the growth depression).In fact, it would be surprising if growth quiescence and recovery would not involve also changes in cell wall composition. In addition, 24 hours, the time after treatment when cell walls are extracted, is well after growth recovery sets in, according to the authors' time lapse analysis. Another caveat is that cell wall material will come largely from non-growing material, therefore it is also possible that NaCL stress triggers a change in extractability of different cell wall species.

-EGCG, as all condensed tannins (possibly polyphenols in general), interacts with the cell wall. particularly with pectins. Therefore, its alleviating effect might be structural, rather than enzymatical. This also holds a caveat for the interpretation of the pectin extraction experimnents. In order to show that the effect is through PME activity. In addition, EGCG triggers the activation of BR signalling, which coudl affect response to NaCl as well, for example by controlling cell wall remodelling enzyme expression. In this context, it might be of relevance that whereas the the 2F4 antibody recognizes Ca2+-crosslinked HG, it does not technically show that these crosslinks occur in the wall, as calcium is possibly lost during extraction and the 2F4 buffer contains 0.5 mM CaCl. Therfore, it is better thought of as an AB that detects pectin that can be crosslinked.

-If i understand correctly, the authors' model implicates a direct effect of NaCl, and a counteracting effect of Calcium on the activity of enzymes such as the PMEs which would then trigger cell wall changes, which the would trigger salt responses through the CrRLK1s and MPKs. Consistent with this model, the PME inhibitor EGCG has an alleviating effect when applied together with NaCL. As discussed above, other explanations are possible; thus, to support their model PME activities (in vitro) could be assessed.

-It is very difficult to establish causality between the early salt effects such as gene expression and the late effects such as survival or changes in growth. In several instances, causality is implied in the MS, without strong evidence in favor of this causality.

-In my reading (caveats apply), the idea behind the original model by Feng et al., is slightly mis-characterized and i am not sure the author's data aren't also consistent with that earlier model. I was under the impression that the idea was that Na ions displace Ca ions from the eggbox pectin and that it is this egg box dissociation which is recognized. Thus, the eggboxes would act as a "guarded" signalling epitope under surveillance to indicate ionic stress. At the same time, there might of course also structural implication of this displacement (e.g. swelling) which might also be recognized. Ca ions could alleviate the structural integrity problems even in the absence of CrRLK1-mediated signalling.

-At least one PME mutant has been described to be salt hypersensitive (PMID: 29307824), opposite to what the authors' model would suggest. There are many PME isoforms with many possible

© 2022. Published by The Company of Biologists under the terms of the Creative Commons Attribution License (https://creativecommons.org/licenses/by/4.0/).

functions (plus possible (hyper-)compensatory responses) so that is not strong evidence against the model but i think the study should be mentioned.

-Some PMEs are described to require a certain salt concentration for activity, maybe worth discussing

-The phrase "..THE1-4 is required for.." is, although correct in a strictly genetic sense, a bit odd, considering that the1-4 is a gain-of-function mutant. Thus THE1-4 is implied to have a physiological role here -which it doesn't have

-Figure S4A does not accurately depict the binding epitopes of the pectin antibodies.

Resubmission

First decision letter

MS ID#: DEVELOP/2021/200363

MS TITLE: Arabidopsis root responses to salinity depend on pectin modification and cell wall sensing

AUTHORS: Nora Gigli-Bisceglia, Eva van Zelm, Wenying Huo, Jasper Lamers, and Christa Testerink

I have now received all the referees' reports on the above manuscript, and have reached a decision. The referees' comments are appended below, or you can access them online: please go to BenchPress and click on the 'Manuscripts with Decisions' queue in the Author Area.

As you will see, the referees express considerable interest in your work, but have some significant criticisms and recommend a substantial revision of your manuscript before we can consider publication. If you are able to revise the manuscript along the lines suggested, which may involve further experiments, I will be happy receive a revised version of the manuscript. Your revised paper will be re-reviewed by one or more of the original referees, and acceptance of your manuscript will depend on your addressing satisfactorily the reviewers' major concerns. Please also note that Development will normally permit only one round of major revision.

We are aware that you may be experiencing disruption to the normal running of your lab that make experimental revisions challenging. If it would be helpful, we encourage you to contact us to discuss your revision in greater detail. Please send us a point-by-point response indicating where you are able to address concerns raised (either experimentally or by changes to the text) and where you will not be able to do so within the normal timeframe of a revision. We will then provide further guidance. Please also note that we are happy to extend revision timeframes as necessary.

Please attend to all of the reviewers' comments and ensure that you clearly highlight all changes made in the revised manuscript. Please avoid using 'Tracked changes' in Word files as these are lost in PDF conversion. I should be grateful if you would also provide a point-by-point response detailing how you have dealt with the points raised by the reviewers in the 'Response to Reviewers' box. If you do not agree with any of their criticisms or suggestions please explain clearly why this is so.

Reviewer 1

Advance summary and potential significance to field

In this article, the authors explore the response to the salt of Arabidopsis roots. They first compare the response to salt in mutant in the transmembrane receptor fer and the double mutant thes1/herk1. They demonstrate that these mutants have an enhanced early response (minutes to hours) using described genetic reporters. This early response is associated with the stronger long-term salt stress response such as root angle and root growth. To correlate this phenotype with

© 2022. Published by The Company of Biologists under the terms of the Creative Commons Attribution License (https://creativecommons.org/licenses/by/4.0/).

changes in cell wall structure they show that the salt treatment is associated with changes in methylation in the extractable pectin as it was demonstrated in the past (Nari Noat Ricard 1991 Biochem). This increase is stronger in the two mutants than in the wild type but is also visible in the mutant in salt signaling mpk6-3. Finally, they confirm the link between HG pectin chemical changes with slate sensor response by combining Ca and PME inhibitor to salt stress. These results are then discussed and interestingly integrated into the known data on HG sensing.

This work confirms the importance of HG metabolism in short and long-term responses to salt stress and the need for the functional transmembrane receptor for a proper response. It confirms the effect of salt on PME activity and the specific function of Na+ ions in this process.

Comments for the author

My main concern is in tune with the discussion of the authors: this work lacs the cytological data. As expressed by the authors it is crucial to separate the tissue specificity of the salt response in the different mutants. Without the cellular resolution of changes in HG it is impossible to interpret the results in a simple signaling network as proposed by the authors. The risk is to create an illusion of comprehensive data.
I understand that semiquantitative cytological imaging of HG is challenging but it will greatly increase the impact of this paper. In the fer mutant, most of the response is concentrated on the elongating zone and the early differentiation of the peridermal cells into root hears. I would suggest concentrating on this tissue. Yet the SOS mutant seems to indicate that the endoderm tissues are also crucial for salt resistance indicating that it is potentially in this tissue that HG metabolism undergoes regulation.
I would strongly advise in this work to be particularly attentive to the potential difference in HG chemistry before the salt treatment. Potentially HG changes in some tissue are very likely in the thes1/herk1 before the salt treatment. Such an observation will of course further complicate the interpretation of the results but could be crucial in a future integrative image of the HG implication in the stress response increases the importance of this work.
In conclusion, this very good work brings clarity to the genetic relationship between different elements implicated in the salt stress response. On the other hand, this work does not bring information into the cytological (cellular level) process of the salt response. This imbalance makes it difficult to introduce this data into a comprehensible signaling pathway to salt response and into a comprehensible vision of the physiological processes involved.
For these reasons it is difficult for me to accept the publication of this work as it stands.
I would suggest adding cytological data on HG cartography using triple immunostaining concentrating on the elongating zone at the early salt response in the mutant and with Ca and EGCG treatment. Unfortunately, I can predict that the number of the biological repetitions will have to be important as the HG methylation status is very variable from cell to cell and from plant to plant. I would advise limiting to the minimum all potential elements that could bring variability (light, time of harvest, growing media, the exact position of the tissue studied…).

Reviewer 2

Advance summary and potential significance to field

The authors addressed my concerns and performed new experiments. Importantly, they now show a very early effect of salt on pectin modification which sheds line on the previously unclear order of events. In addition, the authors now provide also PME activity data in Figure 4B where they test the influence of the presence of salt and PME inhibitors in the activity assay. The data are interesting and should be reported, but I am still not convinced they sufficiently support the model. It seems to me that, to justify the conlusions, more solid genetic or biochemical data are needed or the connclusions have to be toned down significantly.

Comments for the author

-The authors suggest that FER responds to salt-induced PME activity or the consequences of PME activity (detectable after ~1 hour) by inhibiting MPK6 activation that is independently triggered by an unknown signal (question mark in the model). The result of this is that the mid-to long-term

© 2022. Published by The Company of Biologists under the terms of the Creative Commons Attribution License (https://creativecommons.org/licenses/by/4.0/).

consequences (gene expression, growth) are alleviated. However, if this is the case, why does 15 minute treatment with salt in the fer-4 and herk1 the1-4 mutants lead to exaggerated MPK6 activity?
-Another unexplained discrepancy is that short term CaCL2 treatment seems to reduce MPK activity and sometimes marker gene expression below mock levels in the absence of NaCl, while EGCG does not have this effect.
-While Figure 4B is a nice experiment, these substances might still work on the substrate, i.e. the pectin provided in the assay (CaCl2, for example, might increase crosslinks, blocking access of PME etc.; see also my previous comments regarding EGCG), One experiment that could solidify the model is to determine extractable PME activity in NaCl-treated vs mock-treated plants. In case PMEs are transcriptionally upregulated by NaCl, one would expect to observe increased activity in the assay under comparable conditions. Of course, NaCL could also directly increase PME activity biochemically, in which case extractable activity should be similar.
-Discussion and Introduction are rather lengthy but some parts are repetitive between the two. On the other hand, relevant literature regarding pectin sensing is missing. There is also a new study by Zhenbiao Yang's group (Lin et al., 2021, Current Biology) demonstrating pectin binding of FER (supporting Feng 2018) and, importantly, showing downstream signalling consequences of that interaction.
-Are there PMEs that are transcriptionally induced by NaCl (expression databases)?

First revision

<u>Author response to reviewers' comments</u>

Reviewer 1 Advance Summary and Potential Significance to Field:
In this article, the authors explore the response to the salt of Arabidopsis roots. They first compare the response to salt in mutant in the transmembrane receptor fer and the double mutant thes1/herk1. They demonstrate that these mutants have an enhanced early response (minutes to hours) using described genetic reporters. This early response is associated with the stronger long-term salt stress response such as root angle and root growth. To correlate this phenotype with changes in cell wall structure they show that the salt treatment is associated with changes in methylation in the extractable pectin as it was demonstrated in the past (Nari Noat Ricard 1991 Biochem). This increase is stronger in the two mutants than in the wild type but is also visible in the mutant in salt signaling mpk6-3. Finally, they confirm the link between HG pectin chemical changes with slate sensor response by combining Ca and PME inhibitor to salt stress. These results are then discussed and interestingly integrated into the known data on HG sensing.This work confirms the importance of HG metabolism in short and long-term responses to salt stress and the need for the functional transmembrane receptor for a proper response. It confirms the effect of salt on PME activity and the specific function of Na+ ions in this process.

Reviewer 1 Comments for the Author:
My main concern is in tune with the discussion of the authors: this work lacs the cytological data. As expressed by the authors it is crucial to separate the tissue specificity of the salt response in the different mutants.
Without the cellular resolution of changes in HG it is impossible to interpret the results in a simple signaling network as proposed by the authors. The risk is to create an illusion of comprehensive data.
I understand that semiquantitative cytological imaging of HG is challenging but it will greatly increase the impact of this paper. In the fer mutant, most of the response is concentrated on the elongating zone and the early differentiation of the peridermal cells into root hears. I would suggest concentrating on this tissue. Yet the SOS mutant seems to indicate that the endoderm tissues are also crucial for salt resistance indicating that it is potentially in this tissue that HG metabolism undergoes regulation. I would strongly advise in this work to be particularly attentive to the potential difference in HG chemistry before the salt treatment. Potentially HG changes in some tissue are very likely in the thes1/herk1 before the salt treatment. Such an observation will of course further complicate the interpretation of the results but could be crucial in a future integrative image of the HG implication in the stress response increases the importance of this

© 2022. Published by The Company of Biologists under the terms of the Creative Commons Attribution License (https://creativecommons.org/licenses/by/4.0/).

work. In conclusion, this very good work brings clarity to the genetic relationship between different elements implicated in the salt stress response. On the other hand, this work does not bring information into the cytological (cellular level) process of the salt response. This imbalance makes it difficult to introduce this data into a comprehensible signaling pathway to salt response and into a comprehensible vision of the physiological processes involved.

For these reasons it is difficult for me to accept the publication of this work as it stands. I would suggest adding cytological data on HG cartography using triple immunostaining concentrating on the elongating zone at the early salt response in the mutant and with Ca and EGCG treatment. Unfortunately, I can predict that the number of the biological repetitions will have to be important as the HG methylation status is very variable from cell to cell and from plant to plant. I would advise limiting to the minimum all potential elements that could bring variability (light, time of harvest, growing media, the exact position of the tissue studied…).

Response 1.

We understand the concerns of this reviewer and we would like to thank her/him for being so critical and cautious when it comes to understanding the effect of HG modifications in response to salt stress. We do agree with the reviewer that it is important not to create an illusion of comprehensive understanding of the signaling networks involved without having tissue-resolution data. We have tried to capture our results in a model, but also realize that a model always needs to simplify and summarize in order to remain informative. We very much agree with both reviewers that the signaling pathways we have uncovered in this work are likely not linear and simple and might differ between different tissues. We therefore modified the figure legend of the model by adding another sentence to the figure legend to emphasize this point (see New Figure 6). In our work, we do report for the first time that PME activation is altered when salt is present. Additionally, as now shown in New Figure S4 (and response to reviewer 2) this does not depend on a differential transcriptional activation of the PMEs but seems to be determined by a direct effect that salt has on the PME activity. Indeed, it is possible that the PME distribution differs between tissues, and that this event would alter the levels of de-methylated HG in different seedling regions. To completely answer the question in "which tissue the reported pathways act", it would require not only cell wall labeling (and HG cytology) but also PME activity and distribution in the apoplast of different tissues, and actually also localization of all other players reported, such as the receptor kinases and MPK6. This is one of the reasons why we did most of the analysis of this paper (like the intracellular responses as well as the cell wall analysis) in whole seedlings without making a distinction between cell layers or focusing on a specific tissue. We think that only by associating tissue specific- PME activity and cell wall histology it would be possible to link directly salt to cell wall modifications, but in our opinion, the effort required to address this question would be beyond the scope of the present manuscript. **Please see modified Figure 6 legend and new Figure S4.**

Reviewer 2 Advance Summary and Potential Significance to Field:
The authors addressed my concerns and performed new experiments. Importantly, they now show a very early effect of salt on pectin modification which sheds line on the previously unclear order of events. In addition, the authors now provide also PME activity data in Figure 4B where they test the influence of the presence of salt and PME inhibitors in the activity assay. The data are interesting and should be reported, but I am still not convinced they sufficiently support the model. It seems to me that, to justify the conlusions, more solid genetic or biochemical data are needed or the connclusions have to be toned down significantly.

Reviewer 2 Comments for the Author:
Comment 1_*The authors suggest that FER responds to salt-induced PME activity or the consequences of PME activity (detectable after ~1 hour) by inhibiting MPK6 activation that is independently triggered by an unknown signal (question mark in the model). The result of this is that the mid-to long-term consequences (gene expression, growth) are alleviated. However, if this is the case, why does 15 minute treatment with salt in the fer-4 and herk1 the1-4 mutants lead to exaggerated MPK6 activity?*

© 2022. Published by The Company of Biologists under the terms of the Creative Commons Attribution License (https://creativecommons.org/licenses/by/4.0/).

Response 1. We agree with this reviewer, and we think that it is not simple to discuss the role of the CW sensors in regulating stress responses in the presence and absence of salt. We have no evidence that would support the hypothesis that in the absence of these proteins, the cell wall composition might be altered. However, this has been suggested in different other papers, starting from *Feng et al 2018* where the authors show through AFM analysis that in the presence of salt, *fer-4* mutants show softer cell walls leading to severe cell swelling that can be mitigated by either calcium or borate application. Preexisting cell wall defects might explain the differences in the intensity of the responses that are observed in the presence of salt. If we hypothesize that the absence of the sensors changes cell wall composition, we could assume that the enhanced levels of the marker gene expression as well as MPK6 activity, observed in controls, can be explained by a preexisting damage that can be alleviated by the presence of chemicals that directly or indirectly alleviate the damage. This is true for calcium (and for borate) but it also seems to be (even if in a more subtle manner) visible when EGCG is applied. In fact when calcium is applied alone, the already evident MPK6 activation or the expression of the selected marker genes (observed for example in control treated *fer-4*) decreases, suggesting that the inhibition of the cell wall damage through the application of chemicals that can create cell wall crosslinks (and reinforce the cell wall) is already sufficient to partly switch off these responses. Given that the intensity of many intracellular responses in response to salt is stronger in the cell wall sensor mutants, we can speculate that being the cell walls of these mutants already altered, responses to chemicals (like salt) that really have an impact on the cell wall composition and architecture can lead to a stronger damage. In this scenario one branch of the responses might depend on a direct inhibition mediated by the cell wall sensors of the downstream responses, while the other (showed with an independent arrow that goes from the cell membrane to MPK6) might depend purely on cell wall damage, likely being uncoupled to the active function of the cell wall sensors but still dependent on their effect on the physiology of the cell walls. To summarize we think that this discrepancy can be only tackled if we hypothesize that mutants for the cell wall integrity alters the cell wall structure leading to cell wall damage. This results in them being more sensitive to salt stress (as we show in all the figures) and the effect of salt is stronger than in the wt being the consequence of their basal stress and the applied salt stress. This means that the parallel branch that goes from salt to MPK6 (which we draw in figure 6 as a question mark) is independent on the presence of the cell wall sensors *per se*, but might be dependent on their role in indirectly altering the cell wall composition leading to the activation of certain damage responses that can be alleviated by chemicals that reinforce the cell walls. We now have included this consideration in the discussion section, but also in the legend of Figure 6.
Please see modified Figure 6 legend and discussion.

Comment 2_*Another unexplained discrepancy is that short term CaCL2 treatment seems to reduce MPK activity and sometimes marker gene expression below mock levels in the absence of NaCl, while EGCG does not have this effect.*

Response 2. We thank this reviewer because we realized that this point was not discussed at all in the manuscript. We think that calcium might have a faster and in general stronger effect than EGCG. First because, as also suggested by this reviewer and has reported in several papers, $CaCl_2$ in solution releases calcium ions which are hypothesized to intercalate in the cell wall, likely enhancing cell wall links. In this scenario mutants altered in their cell wall integrity can be alleviated by calcium application (as reported for the FER-dependent root phenotypes in Feng et al 2018) and as shown in this paper reducing basal gene expression levels and the physiological MPK6 activation altered in the CWI mutants. Moreover, the effect of calcium seems stronger when compared to EGCG in terms of its ability to inhibit PME activity. We present this result in Figure 4B, where we show that at the concentration used in this paper, $CaCl_2$-dependent PME inhibition is statistically greater when compared to EGCG. Thus, we suggest that the reason why we observe differences between CaCl2 and EGCG might be due to the dual effects of calcium ions on both PME activity and cell wall crosslinks, which is turn might result in a faster and stronger alleviation of the salt stress phenotypes. This part has been included in figure 6's legend as well as in the discussion section. **Please see modified Figure 6 legend and discussion.**

Comment 3_*While Figure 4B is a nice experiment, these substances might still work on the substrate, i.e. the pectin provided in the assay (CaCl2, for example, might increase crosslinks, blocking access of PME etc.; see also my previous comments regarding EGCG), One experiment that could solidify the model is to determine extractable*

© 2022. Published by The Company of Biologists under the terms of the Creative Commons Attribution License (https://creativecommons.org/licenses/by/4.0/).

PME activity in NaCl-treated vs mock-treated plants. In case PMEs are transcriptionally upregulated by NaCl, one would expect to observe increased activity in the assay under comparable conditions. Of course, NaCL could also directly increase PME activity biochemically, in which case extractable activity should be similar.

Response 3. We have performed this experiment by analyzing the PME activity in protein extracts of wt seedlings treated with mock, NaCl 100 mM, EGCG or NaCl/EGCG for 3, 6 and 24 h. We spotted the same amount of proteins and analyzed the PME activity as in Figure 4B (by determining the area in cm^2). As shown in NEW Figure S4B no statistically significant difference was detected, nor between the time points or between the treatments. This experiment was the first experiment we performed. Because we were not sure whether we were losing some subtle effects of these chemicals on the PME activity by using this assay, we also used another approach and detected the released of methanol with the formaldehyde/alcohol oxidase assay (according to *Mueller et al 2013*). As reported in NEW Figure S4C, we were also not able to detect any difference at any time points by analyzing the PME activity measured over time (15 minutes) by analyzing the absorbance at 340 nm in 10 ug proteins from extracts derived from seedlings treated for 3 6 or 24h with mock, NaCl, EGCG or NaCl/EGCG. Because the positive control, the PME inhibitor EGCG, did not show an effect in this assay, we hypothesized that the effect of these chemicals (EGCG or NaCl) was lost in the process of extracting PMEs. In fact, when the chemicals were directly supplemented in the medium (Figure 4B), the inhibitory effect of ECGC as well a strong induction in PME activity by NaCl was clear and statistically evident. Collectively this data suggests that not transcription (See Figure NEW S4D) but a direct effect on the catalytic activity is what might cause the differences detected in this paper. As suggested by this reviewer we included the transcriptomic data presented as a heatmap that displays the log fold change between salt and control treated samples at 0.5, 1, 3, 6, 12 and 24 h of *PME* genes (selection of genes according to Wang et al 2013). The microarray dataset which is derived from *Killian et al 2007* clearly shows that in this experimental set up no clear differences in gene expression for any of the PMEs were detected, supporting the idea that the chemicals only have a direct effect on the PME rather than altering the PME pool. **Please see new Figure S4.**

Comment 4_*Discussion and Introduction are rather lengthy but some parts are repetitive between the two. On the other hand, relevant literature regarding pectin sensing is missing. There is also a new study by Zhenbiao Yang's group (Lin et al., 2021, Current Biology) demonstrating pectin binding of FER (supporting Feng 2018) and, importantly, showing downstream signalling consequences of that interaction.*

Response 4. We want to thank this reviewer because we think in fact that this is one of the most relevant papers that connects cell wall integrity sensors and pectin perception. It was not included in the previous versions of the manuscript because it was published after we sent the revision back to this journal. In the new version of the manuscript, we cut the introduction and include relevant literature related to pectin perception *(Lin et al* as well as information regarding pectin sensing such as the role of WAKs/ PERK4) as suggested by this reviewer. We also improved our discussion by adding considerations connected to comments 1 and 2 from this reviewer. We think, that besides modifying Figure 6's legend, it is important that we discuss all relevant considerations and explanations behind the hypothesis drawn in Figure 6 in the discussion section as well. **Please see introduction and discussion section.**

Revised text Figure legend Figure 6:

Figure 6. Hypothetic model based on the findings of this paper. Salt application directly modifies the activity of pectin methyl-esterase (Fig. 4B) triggering cell wall de-methyl esterification (Fig. 3). Detection of these cell wall modifications seems to be responsible, at least in part for the activation of salt stress responses. We show that chemicals that can inhibit PME activity (Fig. 4B) and homogalacturonan methyl-esterification (Fig. 4C, D), can also alleviate the responses to salt stress (Fig 5A, B, C, D). We hypothesize that the cell wall sensors FERONIA (FER) or HERK1/THESEUS1 attenuate most of the salt-dependent phenotypes, being required to negatively regulate the salt-dependent MPK6 activation in response to salt stress (Fig. 1C, D, E). However, because in control conditions these mutants already displayed minor enhanced marker gene expression as well as MPK6 activity (Fig 1C, D, E and 5A, B, C, D), we could speculate that their

© 2022. Published by The Company of Biologists under the terms of the Creative Commons Attribution License (https://creativecommons.org/licenses/by/4.0/).

functional impairment might cause cell wall modifications that can be alleviated by the presence of chemicals that directly or indirectly alleviate the damage (Fig. 4B, C, D). We show that $CaCl_2$ application reduces basal gene expression levels and physiological MPK6 is activation altered in the CWI mutants (Fig 5A, C), likely suggesting that its dual effect on strongly inhibiting PME activity (Fig 4B) and/or cell wall crosslinks, might result in a faster and stronger alleviation of the salt stress phenotypes. On the other hand, responses to chemicals (like salt) that have an impact on the cell wall composition lead to a stronger damage which correlates with a higher intensity of responses in the cell wall sensor mutants. In this scenario one branch of the responses might depend on a direct inhibition mediated by the cell wall sensors of the downstream responses, while the other (displayed as a question mark) might be uncoupled but still be dependent on their effect on the physiology of the cell walls. At the same time, the cell wall sensors seem to only mildly affect halotropism, a root bending response that occurs independently of MPK6 and or $CaCl_2$ application. It should be noted that all the cell wall analyses reported in this paper derive from whole Arabidopsis seedling material and knowledge regarding tissue or cell type where cell wall modifications occur in response to salt remains to be addressed.

Second decision letter

MS ID#: DEVELOP/2021/200363

MS TITLE: Arabidopsis root responses to salinity depend on pectin modification and cell wall sensing

AUTHORS: Nora Gigli-Bisceglia, Eva van Zelm, Wenying Huo, Jasper Lamers, and Christa Testerink
ARTICLE TYPE: Research Article

I am happy to tell you that your manuscript has been accepted for publication in Development, pending our standard ethics checks.

Reviewer 1

Advance summary and potential significance to field

In this manuscript, the authors try to relate the salt stress response and the cell wall integrity pathway in Arabidopsis Thaliana. Indeed, NaCl has a unique ability to rapidly affect roots in this species and is associated with rapid changes in cell wall mechanical properties. The response to these changes is mediated in part through the transmembrane receptors thought to be implicated in cell wall integrity sensing.
This manuscript describes the interaction of transmembrane receptor Feronia, the double mutant Hercules/Theseus, Homogalacturonane chemistry, and salt tolerance.
A full comparison of salt stress response between wild-type plants and the Feronia and Hercules/Theseus mutant is presented including cotyledon bleaching, root growth kinematic, and halotropism. Then the authors present a correlation between salt response and changes in Homogalacuronane methylation on root global extract and pectin methylesterase activity on similar extract. The authors quantify the mediation of this phenotype with changes in pectin methylesterase activity through calcium or pectin methylesterase inhibitor. Finally, the effect of the Feronia Hercules/Theseus regulation on the expression of genes implicated in the stress response is presented.
Thanks to all this work the authors present a more complete vision of the role of Feronia Hercules/Theseus on salt response and halotropism. They further demonstrate the importance of PME and Homogalacturonane metabolism on root response.
One of the exciting observations is the inhibitory effect of Calcium on PME activity both in vitro and in vivo. The authors' observations are in tune with the previous observation from Vincent, R.R.R., (Vincent, R.R.R., Mansel, B.W., Kramer, A., Kroy, K., Williams, M.A.K., 2013. Micro-rheological behavior and nonlinear rheology of networks assembled from polysaccharides from the plant cell wall. New J. Phys. 15, 35002. https://doi.org/10.1088/1367-2630/15/3/035002)

© 2022. Published by The Company of Biologists under the terms of the Creative Commons Attribution License (https://creativecommons.org/licenses/by/4.0/).

Comments for the author

The importance of salt response for agriculture is undeniable and all advances in its understanding are important, in that sense the work presented is interesting. This in all aspects very good work could be published in Development after certain modifications and improvements.
Concerns:
-First as indicated the Ca2+ inhibition of PME activity has been reported previously. Thus the manuscript should state it and cite the appropriate literature.
-Secondly, as indicated by the authors in the discussion this manuscript lacks a cytological description of the effect of salt on the root, especially the Homogalactornane cartography in the root.
As the salt stress provokes accelerated differentiation of cells of the elongating zone into root hairs. I would advise adding a description of this phenomenon for all the plants studied. It is important as some of the mutants studied have root hair formation defects. It is therefore important to report the link between the two phenomena.
I also strongly suggest performing multicolor colored immunohistochemistry to elucidate in which cell wall the salt stress leads to changes in PME activity and HG methylation.

Reviewer 2

Advance summary and potential significance to field

The authors have addressed my concerns to large extent and i acknowledge that it is difficult to significantly go beyond the current experiments. The new PME activity measurements provide rather strong support for the authors' hypothesis that salt directly affects PME activity. The authors also made an effort to carefully discuss the loose ends of the study and are careful not to overstate their conclusions.

Comments for the author

I only have a minor suggestion which is to carefully review the references in the manuscript, especially those related to the cell wall and signalling. For example PME-dependency of polygalacturonases was shown already by Wakabayashi et al.. Wolf et al., 2012 did use EGCG as PME inhibitor but the discovery of that effect of the catechin was made by Lewis et al., 2008, Carbohydrate Research and thus this would be the appropriate reference. The former publication, on the other hand, describes why long-term treatment with EGCG might not be expected to lead to overall altered PME activity, as observed by the authors.

© 2022. Published by The Company of Biologists under the terms of the Creative Commons Attribution License (https://creativecommons.org/licenses/by/4.0/).
